# Comparative Transcriptome Profiling of the Loaches *Triplophysa bleekeri* and *Triplophysa rosa* Reveals Potential Mechanisms of Eye Degeneration

**DOI:** 10.3389/fgene.2019.01334

**Published:** 2020-01-16

**Authors:** Qingyuan Zhao, Renyi Zhang, Yingqi Xiao, Yabing Niu, Feng Shao, Yanping Li, Zuogang Peng

**Affiliations:** ^1^ Key Laboratory of Freshwater Fish Reproduction and Development (Ministry of Education), Southwest University School of Life Sciences, Chongqing, China; ^2^ School of Life Sciences, Guizhou Normal University, Guiyang, China

**Keywords:** cavefish, *Triplophysa rosa*, transcriptome, eye degeneration, lens, photoreceptor

## Abstract

Eye degeneration is one of the most obvious characteristics of organisms restricted to subterranean habitats. In cavefish, eye degeneration has evolved independently numerous times and each process is associated with different genetic mechanisms. To gain a better understanding of these mechanisms, we compared the eyes of adult individuals of the cave loach *Triplophysa rosa* and surface loach *Triplophysa bleekeri*. Compared with the normal eyes of the surface loach, those of the cave loach were found to possess a small abnormal lens and a defective retina containing photoreceptor cells that lack outer segments. Sequencing of the transcriptomes of both species to identify differentially expressed genes (DEGs) and genes under positive selection revealed 4,802 DEGs and 50 genes under positive selection (dN/dS > 1, FDR < 0.1). For cave loaches, we identified one Gene Ontology category related to vision that was significantly enriched in downregulated genes. Specifically, we found that many of the downregulated genes, including *pitx3*, *lim2*, *crx*, *gnat2*, *rx1*, *rho, prph2*, and β|γ-crystallin are associated with lens/retinal development and maintenance. However, compared with those in the surface loach, the lower dS rates but higher dN rates of the protein-coding sequences in *T. rosa* indicate that changes in amino acid sequences might be involved in the adaptation and visual degeneration of cave loaches. We also found that genes associated with light perception and light-stimulated vision have evolved at higher rates (some genes dN/dS > 1 but FDR > 0.1). Collectively, the findings of this study indicate that the degradation of cavefish vision is probably associated with both gene expression and amino acid changes and provide new insights into the mechanisms underlying the degeneration of cavefish eyes.

## Introduction

Cavefish have been the focus of numerous studies owing to their species diversity and common convergent phenotypic characteristics. There are many underground caves in the world that are dark, filled with fresh water, and devoid of photosynthetic activity (Rétaux and Casane, 2013). However, even in these ostensibly barren nutrient-poor environments, an array of organisms, notably ray-finned fishes, manage to survive, and are indeed restricted to these habitats (Culver and Pipan, 2009; Rétaux and Casane, 2013). The cavefish, which are found in all continents except Antarctica, are the most successful troglodytes, exhibiting diversified phylogenetic structure and geographical distribution, and spanning 10 orders and 22 families (Bichuette, 2007; Trajano et al., 2010; Soares and Niemiller, 2013; Behrmann-Godel et al., 2017). This wide distribution suggests multiple instances of independent evolution that have resulted in common troglomorphic characteristics, most notably small, sunken, or entirely absent eyes. However, previous studies have indicated that there are different molecular mechanisms underlying the evolution of the degenerate eyes of these fish.

Among ray-finned fish, species of *Astyanax* are considered valuable cavefish models for comparative research, as they also have surface-dwelling forms (Jeffery, 2001). During early embryonic development, the lens of the subterranean *Astyanax mexicanus* begins to regress before any other eye tissue, suggesting that it might play a regulatory role in eye loss (Jeffery and Martasian, 1998; Strickler et al., 2007a; Jeffery, 2009; Strickler and Jeffery, 2009). Indeed, transplantation experiments using *Astyanax* have provided substantial evidence of the role played by the lens in eye development (Yamamoto and Jeffery, 2000), as has research on the cavefish *Sinocyclocheilus anshuiensis* (Yang et al., 2016). In contrast, *Sinocyclocheilus anophthalmus* appears to possess a different lens-dependent mechanism of eye degeneration (Meng et al., 2013). Despite mechanistic differences, these three species (*A. mexicanus*, *S. anshuiensis*, and *S. anophthalmus*) share many retina-related genes with similar expression patterns. For instance, the eye degeneration-linked cone-rod homeobox (*crx*) and its downstream transcription factors, such as neural retina leucine zipper (*nrl*), orthodenticle homeobox 2 (*otx2*), orthodenticle homeobox 5 (*otx5*), nuclear receptor subfamily 2 group E member 3 (*nr2e3*), G protein subunit alpha transducin 1 (*gnat1*), *gnat2*, and RAR-related orphan receptor B (*rorb*), are downregulated in two or all three of these species (Strickler and Jeffery, 2009; Gross et al., 2013; Meng et al., 2013; Mcgaugh et al., 2014; Yang et al., 2016). However, although such research findings have enabled us to gain an understanding of the mechanisms underlying of cavefish eyes degeneration from the perspective of development and have provided insights regarding the similarities and differences in gene expression regulation, studies to date on Mexican tetra suggest that amino acid sequence changes might also be involved in eye degeneration (Hinaux et al., 2013; Mcgaugh et al., 2014; Casane and Rétaux, 2016; Yang et al., 2016). Therefore, comparative research on another cavefish would be meaningful to better characterize the different evolutionary mechanisms, such as the regulation of gene expression and protein evolution, associated with eye degeneration.

Southwest China, which is noted for its diverse and complex cave and karst habitats, not surprisingly has a rich cavefish fauna (including species of *Sinocyclocheilus* and *Triplophysa*) (Zhao et al., 2011). More than 100 species (of the 197 species worldwide) of *Triplophysa* (Teleostei, Cypriniformes, Nemacheilidae) are present in China, including many surface-dwellers and at least 27 troglodytes that vary in their degree of eye degeneration. *Triplophysa rosa*, which is indigenous to Chongqing, is a typical member of the latter group (Chen and Yang, 2005). Previous studies on *T. rosa* have focused on morphology (Huang, 2012), molecular markers (Zhao et al., 2014), mitogenome sequencing (Wang et al., 2012), karyotype analysis (Niu et al., 2016), and metabolism (Shi et al., 2018). However, there have to date been no studies that have focused on the molecular mechanisms underlying eye degeneration in *T. rosa*. Therefore, in this study, based on a combination of histological and transcriptome analyses, we performed a comparative investigation of *T. rosa* and the closely related surface-dwelling species *Triplophysa bleekeri*, with a view to clarifying the degree of eye degeneration and the genes involved. Our results stand to make a substantial contribution to gaining a more comprehensive understanding of the processes that are under selection during the evolution of cavefish eye regression.

## Materials and Methods

### Samples

**Figure 1:**
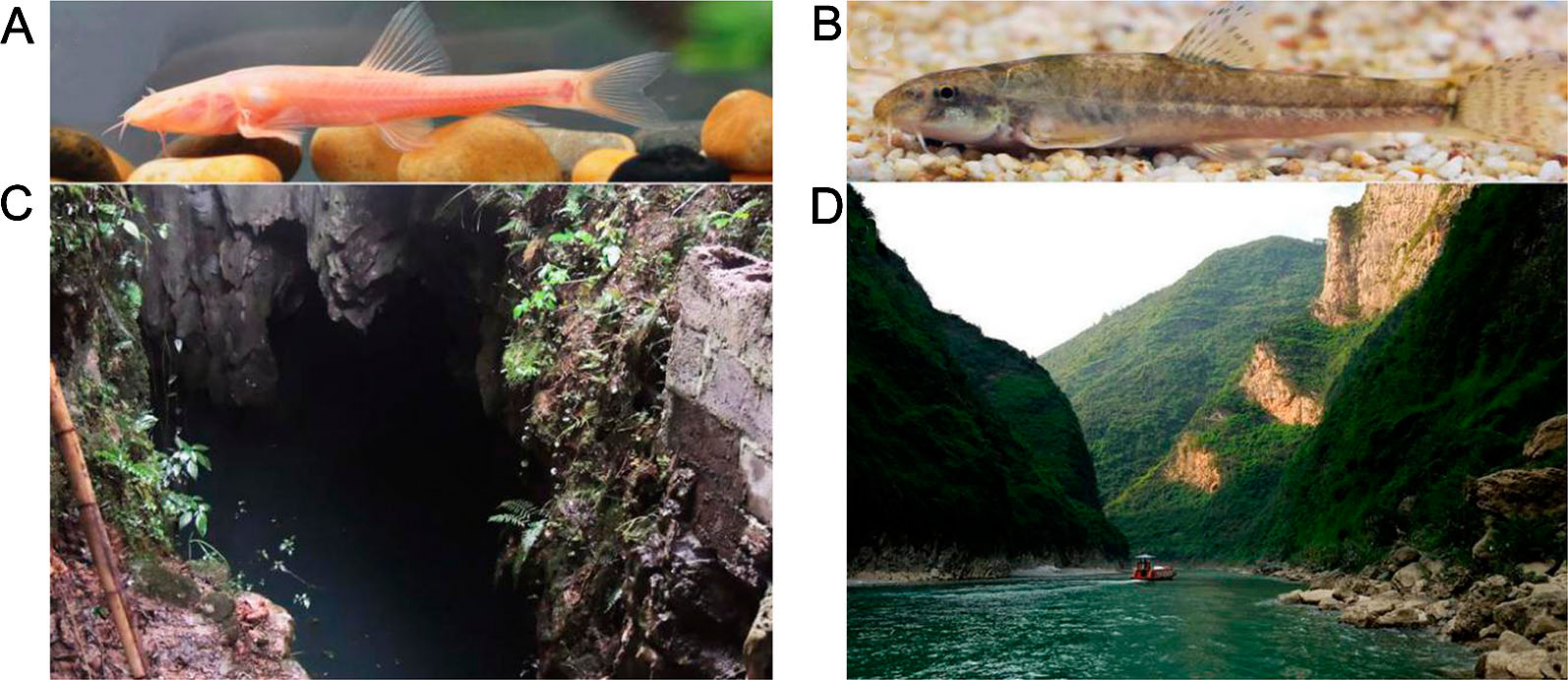
*Triplophysa rosa*, *Triplophysa bleekeri*, and their habitat. **(A)** *T. rosa* (cave loach), **(B)** *T. bleekeri* (surface loach), and their respective habitats: **(C)** a karst cave in Wulong County and **(D)** open rivers system in Wuxi County (Taken by Yabing Niu).

*T. rosa* specimens were collected from Wulong County (**Figures 1A**, **C**) and surface-dwelling *T. bleekeri* were collected from Daling River, Wuxi County (**Figures 1B**, **D**), both of which are in Chongqing, China). In order to replicate conditions in the native habitats as closely as possible, *T. rosa* and *T. bleekeri* specimens were maintained in two separate tanks (140 cm × 160 cm × 80 cm) located in a dark and natural daylight environment, respectively, for 1 week. Water temperature was controlled at 18~20 C using a water cooling device (CW-1000A; Risheng CO., Ltd, Guangdong, China) regulated by a temperature controller (PY-SM5; Pinyi CO., Ltd, Zhejiang, China), and the oxygen concentration was maintained above 7 mg L^-1^ by continuously pumping air using an air pump (HG-750W; Sensen Yuting CO., Ltd, Zhejiang, China). All zoological experiments conducted under approval of the Animal Care and Use Committee of Southwest University.

### Histological and Immunohistochemical Analyses

Adult *T. rosa* and *T. bleekeri* (six individuals each) were euthanized prior to removing their eyes, which were enucleated, cleaned of adipose tissue, and fixed in Bouin's fluid for histological analysis. After 24 h, serial paraffin sections were prepared, and slides were stained with hematoxylin and eosin (H&E), following standard procedures (Li et al., 2014). Cryosectioning and immunostaining of fixed fish eyes (four individuals each) were performed as previously described (Meng et al., 2013). We used monoclonal antibodies against Zpr-1 (ab174435: 1:200; Abcam, Shanghai, China) and 4D2 [fluorescein isothiocyanate (FITC), ab183399: 1:200; Abcam, Shanghai, China] to label red-green cones and rod photoreceptors, respectively. Immunoreactivity was visualized using an Alexa Fluor 488-conjugated anti-mouse IgG secondary antibody (A11017: 1:200; Life Technologies, Eugene, Oregon), except for when staining for 4D2. Slides were premixed with the nuclear stain ToPro3 (T3605: 1:1,000; Life Technologies, Eugene, Oregon) and examined under a NIKON 80i microscope. Fluorescent images were processed using Adobe Photoshop 7.0.

### Library Construction and Illumina Sequencings

Equal proportions of the same tissues (eyes, brain, skin, and gill) of eight individuals of each of the two *Triplophysa* species were initially pooled to provide compound samples, from which total RNA was subsequently extracted using TRIzol Reagent (Invitrogen, Carlsbad, CA). RNA degradation was monitored using 1% agarose gels. The purity and concentration of the extracted RNA were respectively measured using a NanoPhotometer^®^ spectrophotometer (IMPLEN, CA) and a Qubit^®^ RNA Assay Kit in conjunction with a Qubit^®^2.0 Fluorometer (Life Technologies, CA). RNA integrity was assessed using the RNA Nano 6000 assay kit of the Agilent Bioanalyzer 2100 system (Agilent Technologies, CA).

For each pooled sample, aliquots of 3 μg RNA were used as the input material for sample preparations. Sequencing libraries were generated using an NEBNext^®^Ultra™ RNA Library Prep Kit for Illumina^®^ (NEB, Ipswich, MA), following the manufacturer's protocol. Library quality was assessed using the Agilent Bioanalyzer 2100 system. Finally, the four tissue-specific cDNA libraries of each species were clustered and sequenced using the Illumina Hiseq 2500 platform and paired-end reads were generated.

### *De Novo* Transcriptome Assembly and Annotation

Raw data were processed using in-house Perl scripts to remove adapter sequences, reads with ambiguous bases N > 10%, and low-quality reads (> 50% Q ≤ 5 base) (Zhang et al., 2017). Clean reads were *de novo* assembled using Trinity (Grabherr et al., 2011; Haas et al., 2013) with default parameters. Only contigs >200 bp were used for further analysis. For each species, *de novo* reference transcripts were assembled by pooling all four tissue-specific libraries. TGICL-2.1 (Pertea et al., 2003) software was used to reduce the redundancy of the assembled transcripts with an identity threshold of 0.94. Subsequently, the coding region of transcripts was predicted using TransDecoder pipeline (https://transdecoder.github.io), with parameters of -m 100 and -single_best_orf. To provide comprehensive descriptions of the final transcript sets, we employed the Swissprot/Uniprot (Bairoch et al., 2009) and KEGG (Kyoto Encyclopedia of Genes and Genomes) (Kanehisa et al., 2004) public databases, as well as several protein-coding gene sets from the genomes of Cypriniformes species, including those of *Danio rerio* (version GRCz11), *Ctenopharyngodon idella* (version 1), *Sinocyclocheilus grahami* (version 1.1), and *Cyprinus carpio*, to annotate these transcripts.

Cross-species contamination in the resulting transcripts was detected and filtered using CroCo (Simion et al., 2018) with default parameters, and only clean transcripts were used for further analysis. Thereafter, we used CEGMA (Parra et al., 2007) to evaluate the integrity of transcript assembly. The GO annotations were processed using Blast2GO (Götz et al., 2008) for GO database searches based on e-values < 1e-6. To generate a comparative diagram of GO annotation information obtained from the two *Triplophysa* species, we employed OmicShare web software (http://www.omicshare.com/tools) to visualize, compare, and plot the GO annotation results.

### Analysis of Gene Expression and DEGs

Gene expression levels for each sample were estimated using RSEM (Li and Dewey, 2011). The clean reads from the four selected tissues of each species were mapped back onto the species-specific transcripts using Bowtie2 software (Langmead and Salzberg, 2012). Raw read counts per gene were then obtained from the mapping results using RSEM. To compare transcriptional levels between *T. rosa* and *T. bleekeri*, differential expression analysis was narrowed down to only those assembled transcripts that were orthologously presented in the two loaches, as previously described (Jiang et al., 2019; Shu et al., 2019). We used reciprocal best hits (RBH) BLAST (Moreno-Hagelsieb and Latimer, 2008) to identify orthologous genes between the two constitutive transcriptomes. For bidirectional alignment between *T. rosa* and *T. bleekeri*, we used blastn with an e-value 1e-10, and thereafter, transcript pairs with optimal bidirectional alignment and a bit score threshold of ≥300 were identified as orthologous transcripts (Zhang et al., 2015). A scaling normalized factor was used to adjust read counts for each library and differential expression analysis was performed in edgeR (Robinson et al., 2010). The genes corresponding to the transcripts were considered differentially expressed if p-value < 0.01 and |log2FC| > 1. After functional annotation, GO and KEGG pathway enrichment analyses were implemented using the OmicShare web software, both with an FDR < 0.1.

### Identification of Positively Selected Genes (PSGs) and Rapidly Evolving Genes

Sequence sets from six species [*T. rosa* and *T. bleekeri*, the transcriptomes of three loaches or closely related species (*Catostomus commersonii*, *Cobitis taenia*, and *Misgurnus anguillicaudatus*), and one genome gene set of *D. rerio*] were used for analyses of orthologous genes and nonsynonymous (dN)/synonymous (dS) substitution rates (dN/dS), for which data were downloaded from NCBI. Among these, owing to distant multi-species sequencing in *C. commersonii* (Hahn et al., 2016), we detected cross-species contamination and a filter was applied as described. In this study, the term surface loach is used specifically to refer to *T. bleekeri*.

To perform scans on a transcriptome-wide scale for genes under positive selection and obtain dN/dS values for all genes, we first fed the coding sequences of the aforementioned species set along with those of the cave loach branches we wished to examine into the PosiGene pipeline (Sahm et al., 2017). *D. rerio* was used as the PosiGene anchor species. Orthology was determined using PosiGene with RBH BLAST searches (Moreno-Hagelsieb and Latimer, 2008) against the gene set of *D. rerio* (the parameter: -nhs). The dN/dS values and the positive selection genes of *T. rosa* were calculated or detected by codeml (Yang, 2007) in PosiGene. Next, dN/dS values for the surface loach were also calculated by using PosiGene pipeline. Since the PosiGene pipeline only provides final evaluated dN/dS values, to obtain the dN and dS values for all species, it was necessary to recalculate the dN and dS values for each species in order to determine the trends in dN and dS for *T. rosa*. We obtained the final multi-sequence alignment file with genes containing three or more species sequences from the PosiGene pipeline, and subsequently used these orthologs to calculate dN and dS substitution rates using paml-codeml (Yang, 2007) with the same parameter as used for the PosiGene pipeline.

We initially excluded genes with a Ks value greater than two in any branch, due to the possibility of false alignment or pseudogenes (Wang et al., 2019), and performed a comparison of the dN and dS rates among species. To assess potential functional trends dependent on evolutionary rates, we divided our proteins from *T. rosa* (7,275 proteins, as described in Schumacher and Herlyn, 2018) into three equally sized bins of 2,425 proteins according to their dN/dS values as follows: bin 1 (“low rate”; dN/dS ≤ 0.1303); bin 2 (“medium rate”; dN/dS 0.1304–0.3273); and bin 3 (“high rate”; dN/dS ≥ 0.3273). For each bin, we performed an enrichment analysis based on GO and KEGG annotation using OmicShare web software. Further, in order to determine changes in the evolutional trends of vision-related genes dependent on evolutionary rates, we obtained the dN/dS values of all genes involved in each vision-related GO category for comparative analysis between *T. rosa* and *T. bleekeri*.

### Identification of Premature Stop Codons and Frame-Shift Mutations

One-to-one orthologous transcript pairs were derived from the aforementioned results of RBH BLAST between the cave and surface loaches. On the basis of these gene pairs (proteins from the surface loach and transcripts from the cave loach), we then used genewise (Birney et al., 2004) to identify the premature stop codons and frame-shift mutations in the transcripts of the cave loach. The results of candidate pseudogenes were further confirmed using the read alignment results obtained with IGV (Thorvaldsdóttir et al., 2013). Thereafter, we filled the missing bases of the transcripts with frame-shift mutations and re-aligned the reads to these using Bowtie2 (Langmead and Salzberg, 2012). The reads around indels were realigned *via* GATK 3.6 (DePristo et al., 2011) using default parameters. Finally, the sequencing depth of each base was calculated using samtools (http://www.htslib.org/) with parameters -q 20 and -Q 20.

### Quantitative Real-Time PCR Validation of RNA-Seq Data

Given that the eyes of *T. rosa* eyes are very small, to facilitate RNA extraction, tissues from three individual specimens were pooled to form a single sample, whereas only single-individual samples were necessary for *T. bleekeri*. RNA was extracted from three biological replicates (per species) using an RNeasy^®^ plus universal mini kit (QIAGEN, Leipzig, German). Complementary DNA was synthesized using a PrimeScriptTM reverse transcription (RT) reagent kit with gDNA eraser (TaKaRa, Peking, China). Real-time PCR was conducted using SYBR^®^ Premix Ex TaqTM II (TaKaRa, Peking, China). The Tu translation elongation factor, mitochondrial (*tufm*) was used an internal control. Primers were designed based on the relevant genes in both species, derived using AlleleID 6 (Apte and Singh, 2007) from our transcriptome, with synthesis being performed by Invitrogen (see **Table S1** for a list of the primers used). Real-time PCR was conducted using SYBR^®^ Premix Ex TaqTM II (TaKaRa, Peking, China) and an ABI StepOne™ real-time PCR system thermal cycling block.

## Results

### Morphological Differences Between the Eyes of *T. rosa* and *T. bleekeri*

**Figure 2:**
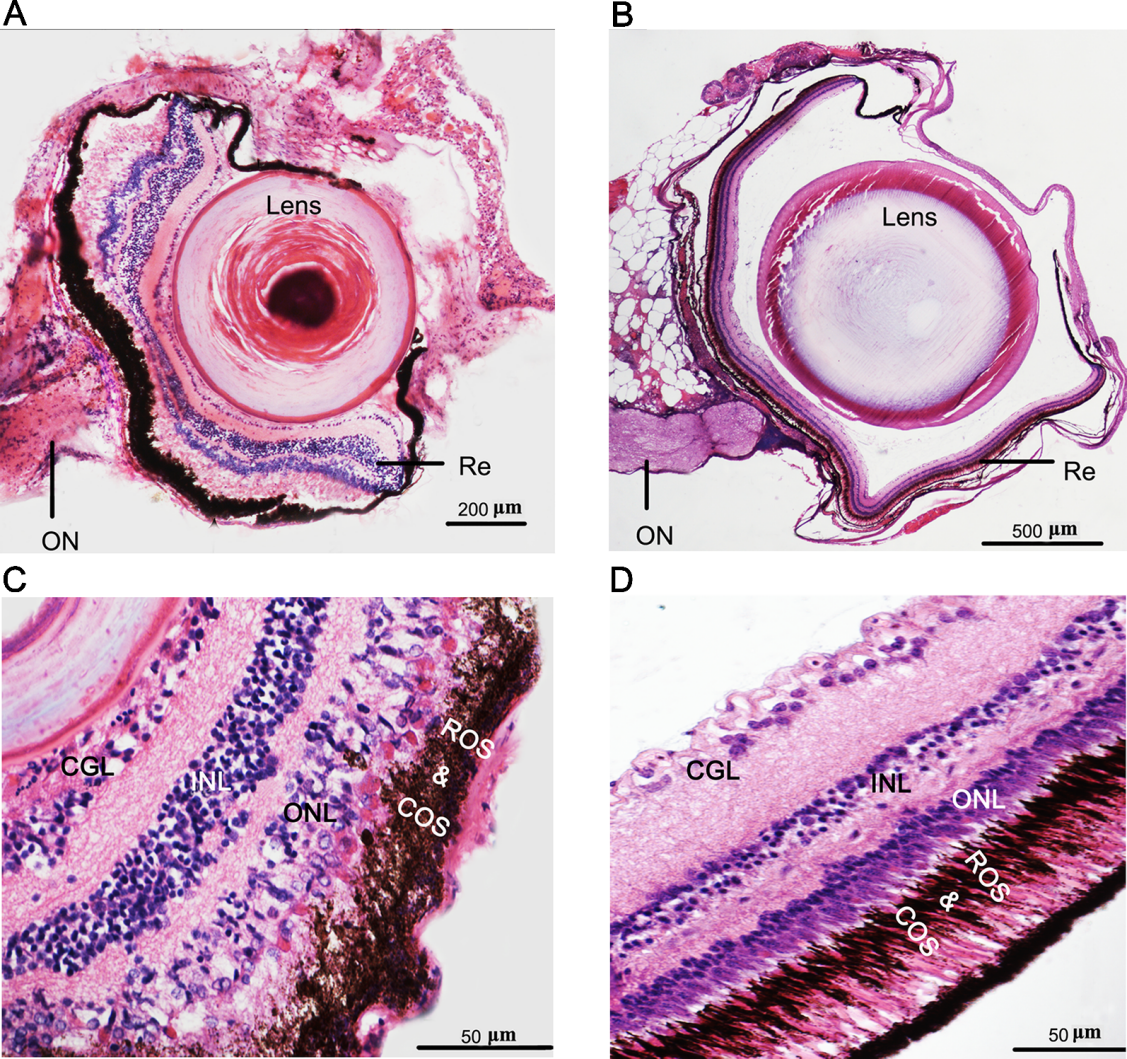
Phenotypes and eye degeneration of cave loach (*Triplophysa rosa*) versus surface loach (*Triplophysa bleekeri*). Stained [hematoxylin and eosin (H&E)] sections of adult *T. rosa* **(A)** eyes and **(C)** retinas, and *T. bleekeri* **(B)** eyes and **(D)** retinas. RE, retina; ON, optic nerve; GCL, ganglion cell layer; INL, inner nuclear layer; ONL, outer nuclear layer; ROS and COS, rod and cone outer segment.

The recessed eyes of *T. rosa* were smaller than the external eyes of *T. bleekeri* (eyeball diameter: *T. rosa* and *T. bleekeri*, 0.50 ± 0.07 and 2.41 ± 0.20 mm, respectively, n = 15 each, **Figures 1A**, **B**). Consistent with this finding, *T. rosa* also possesses smaller optic tecta than those of *T. bleekeri* (Huang, 2012). H&E-stained sections of whole eyes revealed that *T. rosa* has retained the basic vertebrate eye structure, with process lens, cornea, iris, and neural retina. The most distinctive difference between the eyes of the two loaches was that the lens of *T. rosa* has a loose and irregular central portion, whereas the retina exhibits optic nerve atrophy (**Figures 2A**, **B**). Staining revealed that whereas the neural retina of *T. rosa* has retained a layered cellular organization, all three nuclear layers are characterized by pyknotic nuclei, with a larger number of these nuclei clustered in the inner layer. Additionally, the outer nuclear layer was observed to be disordered, indicating that the photoreceptors show greater morphological changes than the inner retinal neurons (**Figures 2C**, **D**).

**Figure 3:**
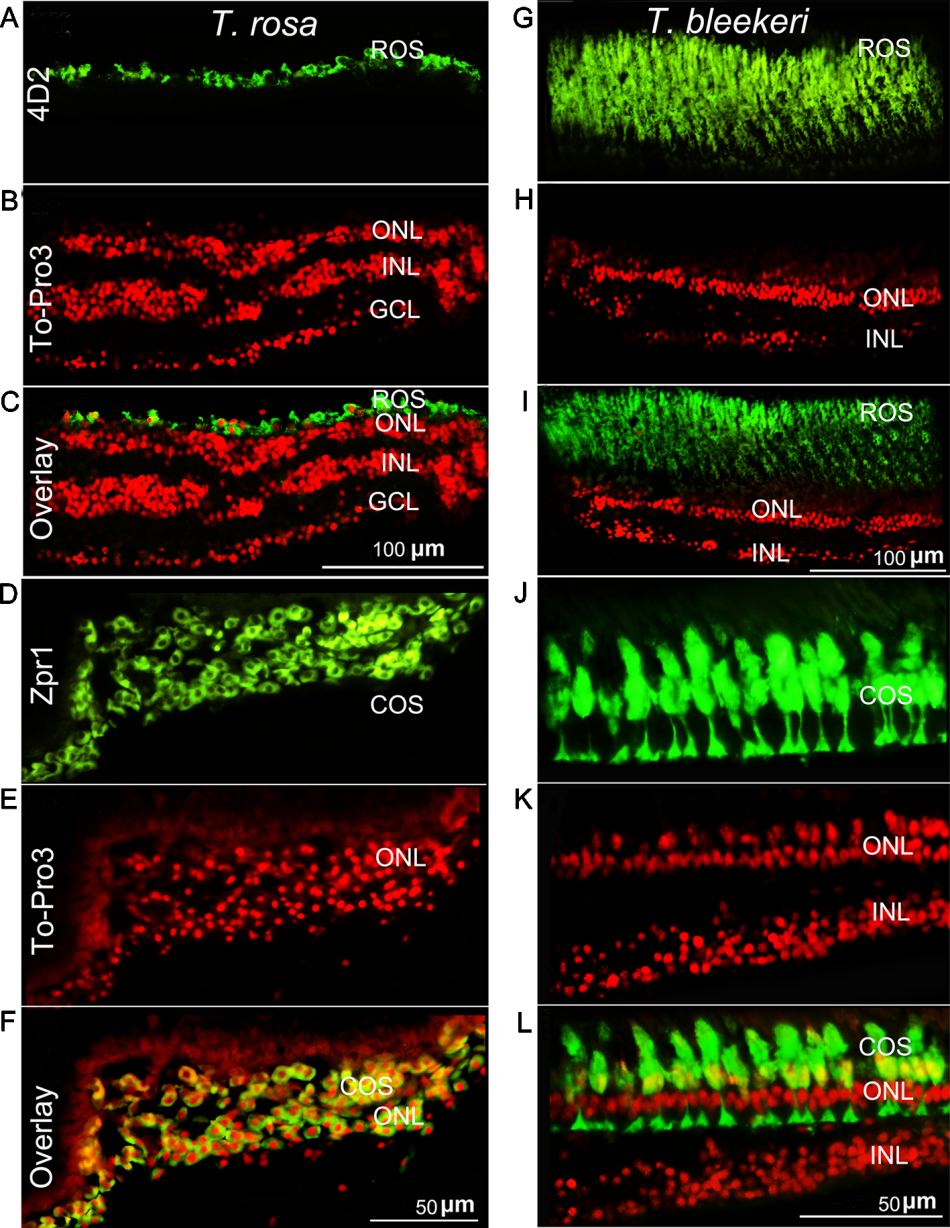
Photoreceptor degeneration in *Triplophysa rosa* and *Triplophysa bleekeri*. Representative double-labeling in retina of **(A–F)** *T. rosa* and **(G–L)** *T. bleekeri*; cell nuclei are labeled red with To-Pro3; outer segments are labeled green. Rod (4D2) and cone (Zpr1) morphology differed between **(A**, **D)** *T. rosa* and **(G**, **J)** *T. bleekeri*. **(C**, **F**, and **I**, **L)** Overlay image showed that the outer segment was only present around the nuclei. RE, retina; ON, optic nerve; GCL, ganglion cell layer; INL, inner nuclear layer; ONL, outer nuclear layer; ROS and COS, rod and cone outer segment.

To further examine differences in the photoreceptor morphology of *T. rosa* and *T. bleekeri* eyes, we labeled the outer segments of the cones and rods with the monoclonal antibodies Zpr-1 and 4D2, respectively, whereas the nuclei were stained with ToPro3. We accordingly observed that the outer segments in *T. rosa* were defective and only present around the photoreceptor nuclei (rod length: *T. rosa* and *T. bleekeri*, 11.96 ± 3.04 and 55.89 ± 8.63 µm, respectively, n = 4 individuals each; cone length: *T. rosa* and *T. bleekeri*, 6.58 ± 3.37 and 39.50 ± 8.03 µm, respectively, n = 4 individuals each). To summarize, *T. rosa* eye degeneration is characterized by an abnormal lens, a retina containing pyknotic nuclei, and substantial morphological abnormalities in photoreceptor structure, along with outer segment deletion (**Figure 3**).

### RNA-Sequencing, Assembly, and Functional Annotation

We generated 275 million raw paired-end reads and 263 million high-quality reads (125 bp). Same assembly methods (**Table 1**) were used to guarantee the acquisition of appropriate transcripts for the two loach species. Total transcript numbers of 105,280 and 92,437 (> 300 bp) were assembled for *T. rosa* and *T. bleekeri,* respectively. On the basis of TransDecoder prediction using several public databases and gene sets, we obtained the final assemblies with 27,922 and 24,454 protein-coding transcripts for *T. rosa* and *T. bleekeri*, respectively (**Table 1**). After filtering with CroCo, we finally obtained 27,316 and 23,255 cleaned protein-coding transcripts for *T. rosa* and *T. bleekeri*, respectively (**Figure S1**). To assess the completeness of the assemblies, we evaluated the two final transcript sets using CGEMA, which revealed that the majority of the 248 eukaryote core genes had been successfully recovered in the two assemblies (**Table S2**). These data thus indicate that the transcripts of the two loaches were well assembled.

**Table 1:** Overview of the *de novo* assembly and annotation of the *Triplophysa rosa* (cave loach) and *Triplophysa bleekeri* (surface loach).

	*T. rosa*				*T. bleekeri*			
	Brain	Skin	Gill	Eye	Brain	Skin	Gill	Eye
Total clean reads	85.5M	88.1M	62.2M	47.5M	51.3M	54.9M	48.1M	88.5M
Total clean nucleotides (nt)	10.68G	11.00G	7.98G	5.94G	6.40G	6.86G	6.02G	11.06G
Numbers of transcripts	105,280				92437			
Mean length (bp)	1054				1132			
N50 length (bp)	1920				2200			
The prediction of CDS	27,922				24,454			
Cleaned transcripts	27,316				23,255			
Annotated in GO	21,476				19,083			
Annotated in KEGG	21,221				18,986			

**Figure 4:**
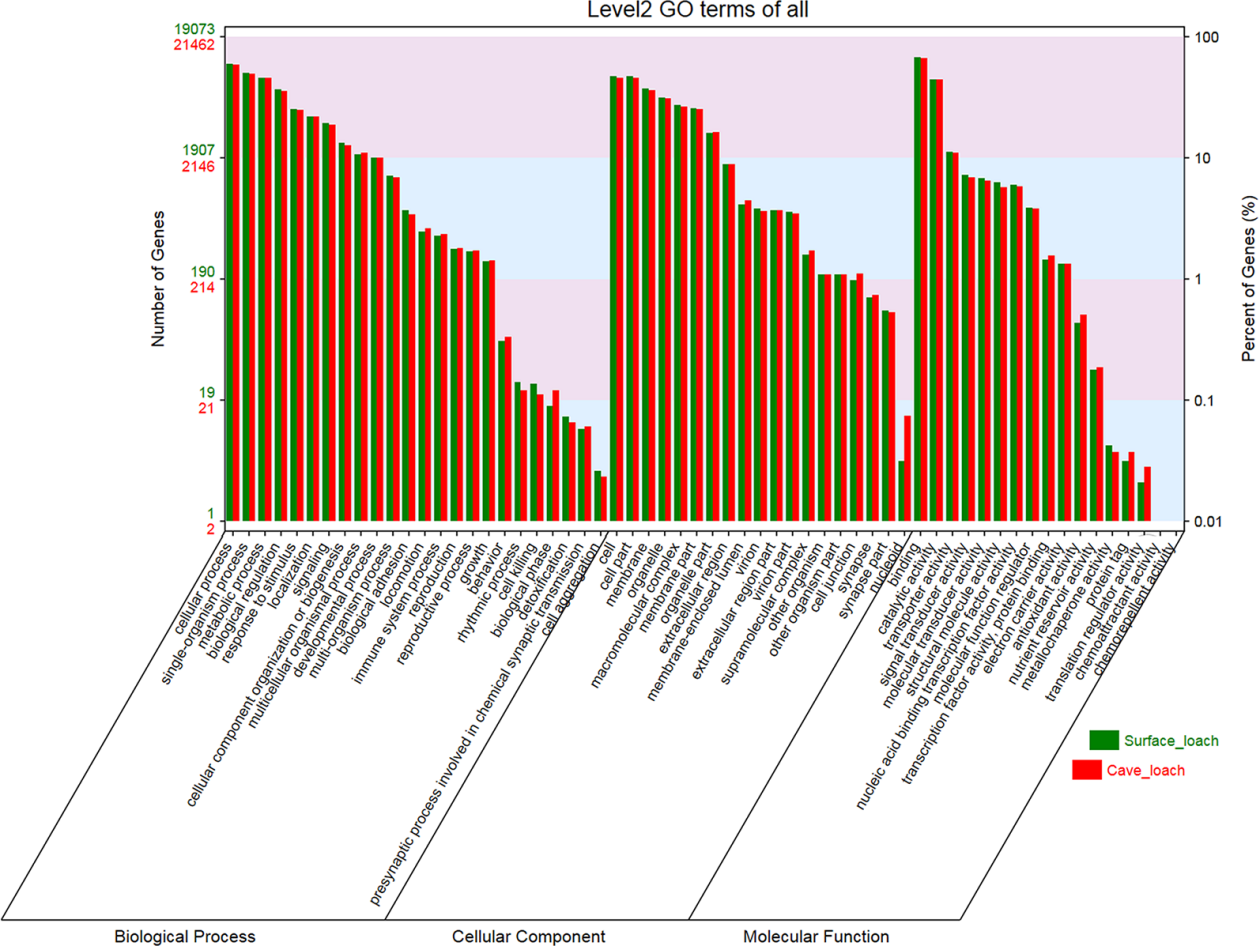
The GO classification of *Triplophysa rosa* (cave loach) and *Triplophysa bleekeri* (surface loach).

On the basis of GO and KEGG database annotations of the two transcript sets (**Table 1**), we obtained a total of 21,476 (78.6%) transcripts mapped to 139,591 GO categories, and 19,083 (82.0%) transcripts mapped to 123,387 GO categories for *T. rosa* and *T. bleekeri*, respectively. The classification of GO categories shows the detailed proportions of individual assemblies, indicating that most categories within molecular function, biological processes, and cellular components were well represented. We also observed a high similarity of GO distribution (r > 0.99, Pearson correlation coefficient) between these two loach species (**Figure 4**).

### Identification and Annotation of DEGs Between *T. rosa* and *T. bleekeri*

To compare differences between the two species at the transcriptional level, we identified 16,325 putative orthologous transcripts between *T. rosa* and *T. bleekeri* based on the RBH method with an e-value cutoff of 1e-10. Furthermore, we identified 4,802 DEGs in the gills (1,244), skin (1,568), brain (1,195), and eyes (3,108). More genes (1,740 upregulated, 1,368 downregulated) were identified as DEGs in the eyes than in other organs, and 2,049 DEGs were specific to the eyes (**Figure S2**).

The 1740 upregulated genes were found to be significantly enriched in 21 GO categories (FDR < 0.1), with the dominant categories being “extracellular region” (187, 10.18%) and “immune system process” (63, 12.55%) (**Table S3**). Cluster analysis of the 1,368 downregulated genes yielded 68 significantly enriched GO categories (**Table S4**), many of which were related to ion channel activity, material transport, and nerve function. Given that vision mainly depends on the formation, transmission, and processing of neural signals, the downregulated expression of ion channels and genes related to neural functions could indicate that cave loach might have lost the necessary material and structural support for vision. Specifically, the GO categories “structural constituent of eye lens” was significantly enriched with the lowest FDR (3.4E-4) in molecular function (**Table 2**). The downregulated expression of genes related to the lens would predictably lead to the abnormal structure of the lens of the cave loach, which is consistent with the aforementioned morphological results.

**Table 2:** Photosensitively related GO categories with low P-value in GO enrichment analysis from down-regulated genes in cave loach.

GO ID	Description	GeneRatio	BgRatio	pvalue	fdr	category
GO:0005212	Structural constituent of eye lens	7	11	3.80E-07	0.00034	Molecular function
GO:0003913	DNA photolyase activity	3	13	0.0305	0.32	Molecular function
GO:0050953	Sensory perception of light stimulus	8	48	0.00376	0.28	Biological process
GO:0048583	Regulation of response to stimulus	55	722	0.00579	0.37	Biological process
GO:0007601	Visual perception	7	46	0.0109	0.50	Biological process
GO:0007423	Sensory organ development	28	332	0.0126	0.56	Biological process
GO:0002088	Lens development in camera-type eye	4	21	0.0239	0.70	Biological process
GO:0002089	Lens morphogenesis in camera-type eye	2	7	0.0507	0.70	Biological process

To identify potential differences in eye development and maintenance in adult cavefish, we focused attention on those genes that are already known to be involved in the development and maintenance of fish eyes. During *T. rosa* lens development and maintenance, we observed downregulation of the transcription factor Paired like homeodomain 3 (pitx3; with log2FC = -4.28), several crystallin genes (e.g. β-|γ-crystallin), and lens intrinsic membrane protein 2 (lim2; with log2FC = -7.42). However, we found αA-crystallin to be absent from both *T. rosa* and *S. anophthalmus*, possibly because the gene is only expressed during early development (Strickler et al., 2007a; Meng et al., 2013).

**Figure 5:**
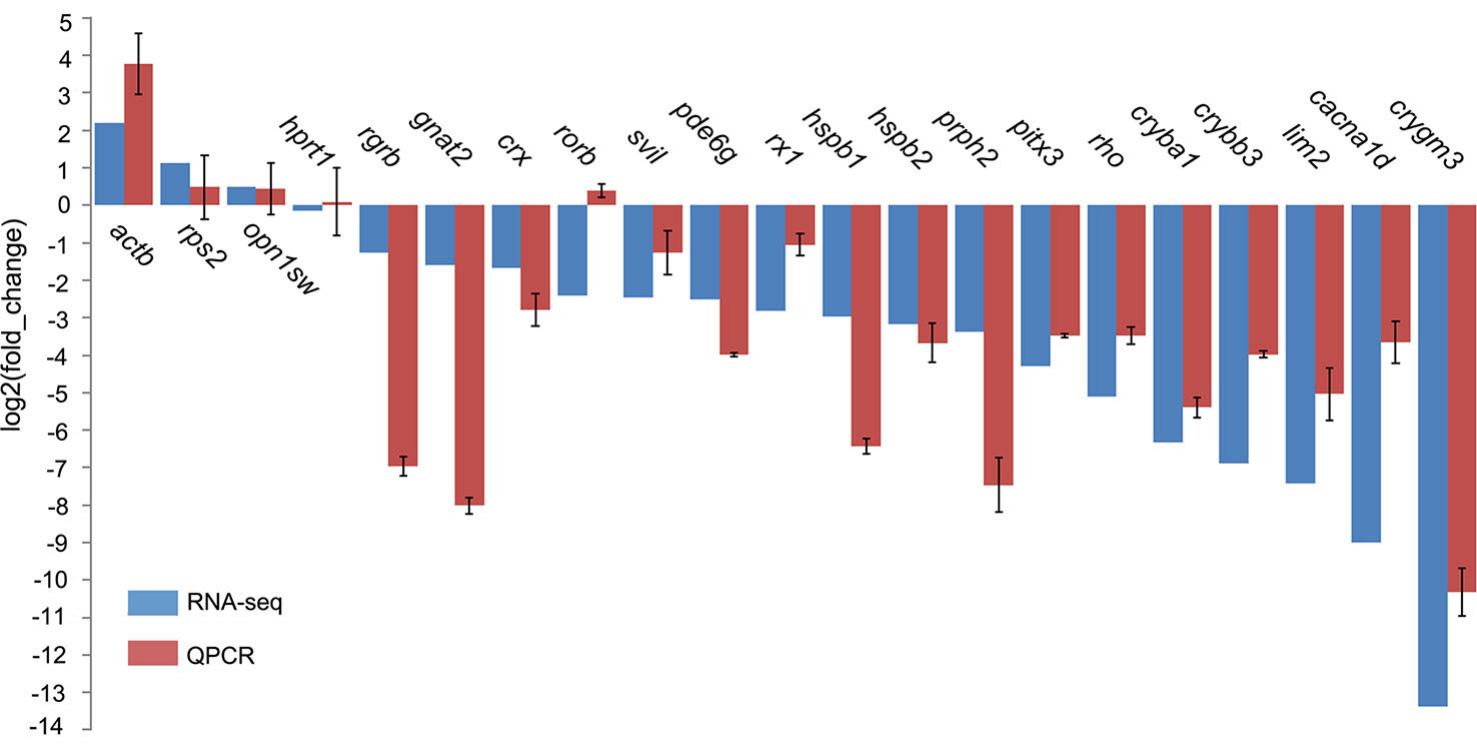
Quantitative polymerase chain reaction (qPCR) validation of selected gene expression in eye tissues. The *tufm* gene was the internal control for expression analyses. Genes *hprt1* and *rps2* were stably expressed and did not significantly differ.

Eye degeneration is generally associated with a vestigial retina, and in this regard, our screening of DEGs involved in retinal cell development revealed four transcription factors (*crx*, *gnat2*, *nr2e3*, and *rx1* ***)***, three photoreceptor genes [*rho*, *rgrb*, and *prph2* (associated with outer-segment disks)], and *pde6g* (associated with visual signal transduction) that were downregulated in *T. rosa*, with degrees of downregulation (expressed as log2FC) of -1.68, -1.59, -2.44, -2.81, -5.12, -1.26, -3.38, and -2.51, respectively (qPCR validated, **Figure 5**). In contrast, we found that the *T. rosa rx2* and *rx3* genes did not show differential expression.

### Analysis of dN, dS, and PSGs

**Figure 6:**
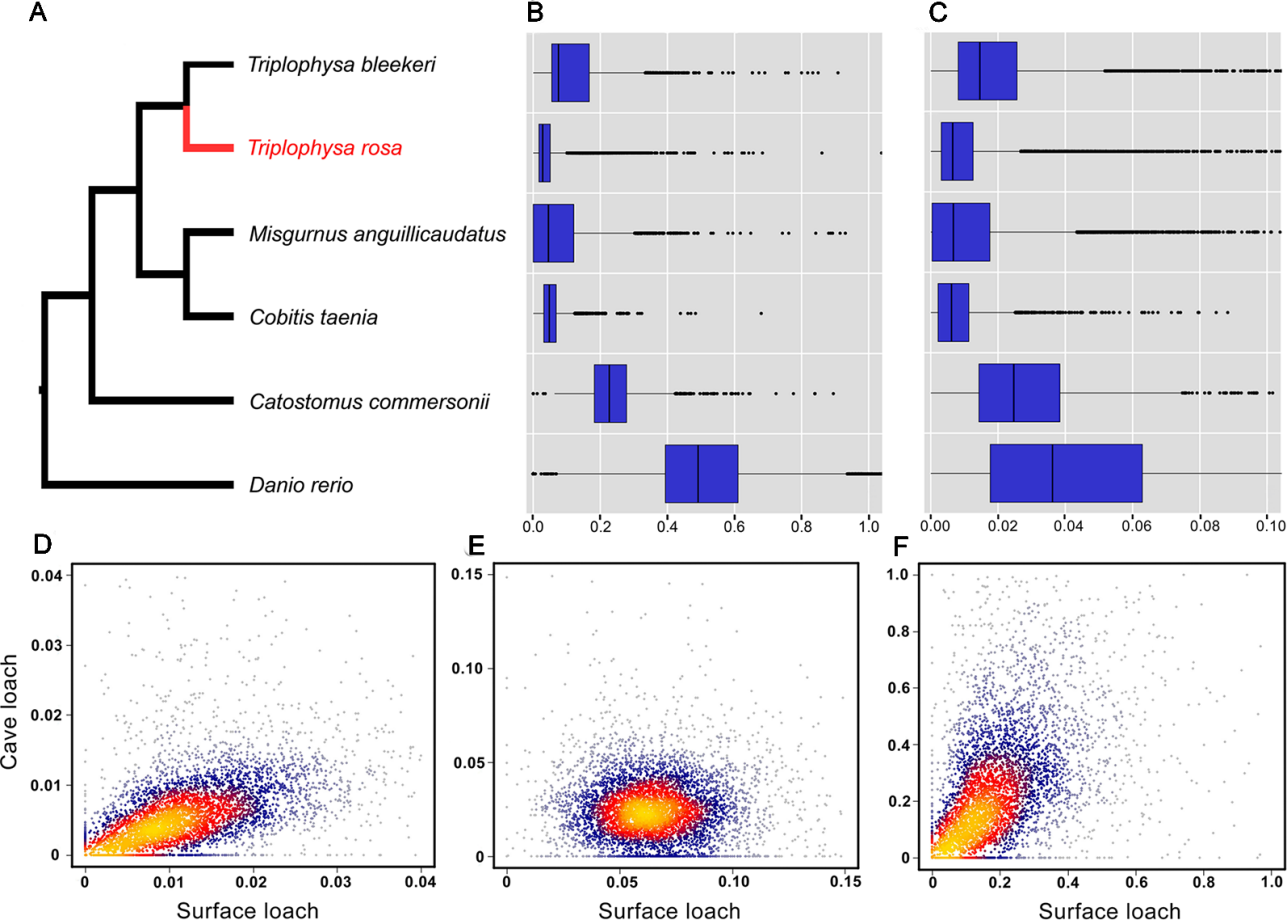
Evolutionary analysis of the cave loach. **(A)** The phylogeny topology of the six Cypriniformes species was reconstructed with posigene pipeline based on orthologues. Comparison of mutation rates in the six fish species based on dS **(B)** and dN **(C)** values. Two-dimension kernel density distribution of dN **(D)**, dS **(E)** and dN/dS **(F)**. The cave loach has much lower Ks values but higher dN values, and so has a much greater dN/dS ratio.

The 10,564 genes expressed by three or more species extracted from the PosiGene pipeline were used for further analysis. Of these, 7,282 genes that are present in cave loach were used to identify genes that have evolved under positive selection *via* the PosiGene pipeline. We obtained 52 PSGs [50 genes: dN/dS > 1; two genes dN/dS < 1, FDR < 0.1 (hold positive selection sites), **Table S5**]; however, the functions of none of these genes is related to vision or light sensitivity. Calculation of the dN and dS values of all species revealed that the dS values of the cave loach were significantly lower than those of its close relative the surface loach (**Figures 6A, B**). Cave loaches also had the lowest dS values relative to those of other species. However, despite the low dS mutation rates for the cave loach (dS, **Figure 6B**), its protein sequences appeared to have a higher evolutional rate (dN rate, **Figure 6C**). Further, a heat scattering plot showed that whereas the dS values for the cave loach were significantly lower than those for the surface loach (**Figures 6A, B**). the dN values of the two loaches were more similar, such that the cave loach showed significantly greater dN/dS values than the surface loach (**Figures 6D**–**F**).

To characterize the evolution of genes associated with the eyes, we assessed potential functional trends in dependence of evolutionary rates. For this, we divided cave loach proteins into three bins based on the dN/dS values and performed GO functional enrichment analysis. To determine the evolutionary trends of vision-related genes, we screened all GO categories related to vision based on the results of GO enrichment, and accordingly found that the GO terms directly related to photosensitivity had a larger number of genes distributed in the high rate bin (**Table S6**). Although some of these GO terms were enriched with a P-value < 0.05, none of the GO categories had an FDR < 0.1. However, genes related to visual development were mainly distributed in the low rate bin. Moreover, we identified two GO categories, “camera-type eye development” and “eye development” that were significantly enriched in the low rate bin with an FDR < 0.1 (**Table S6**).

**Figure 7:**
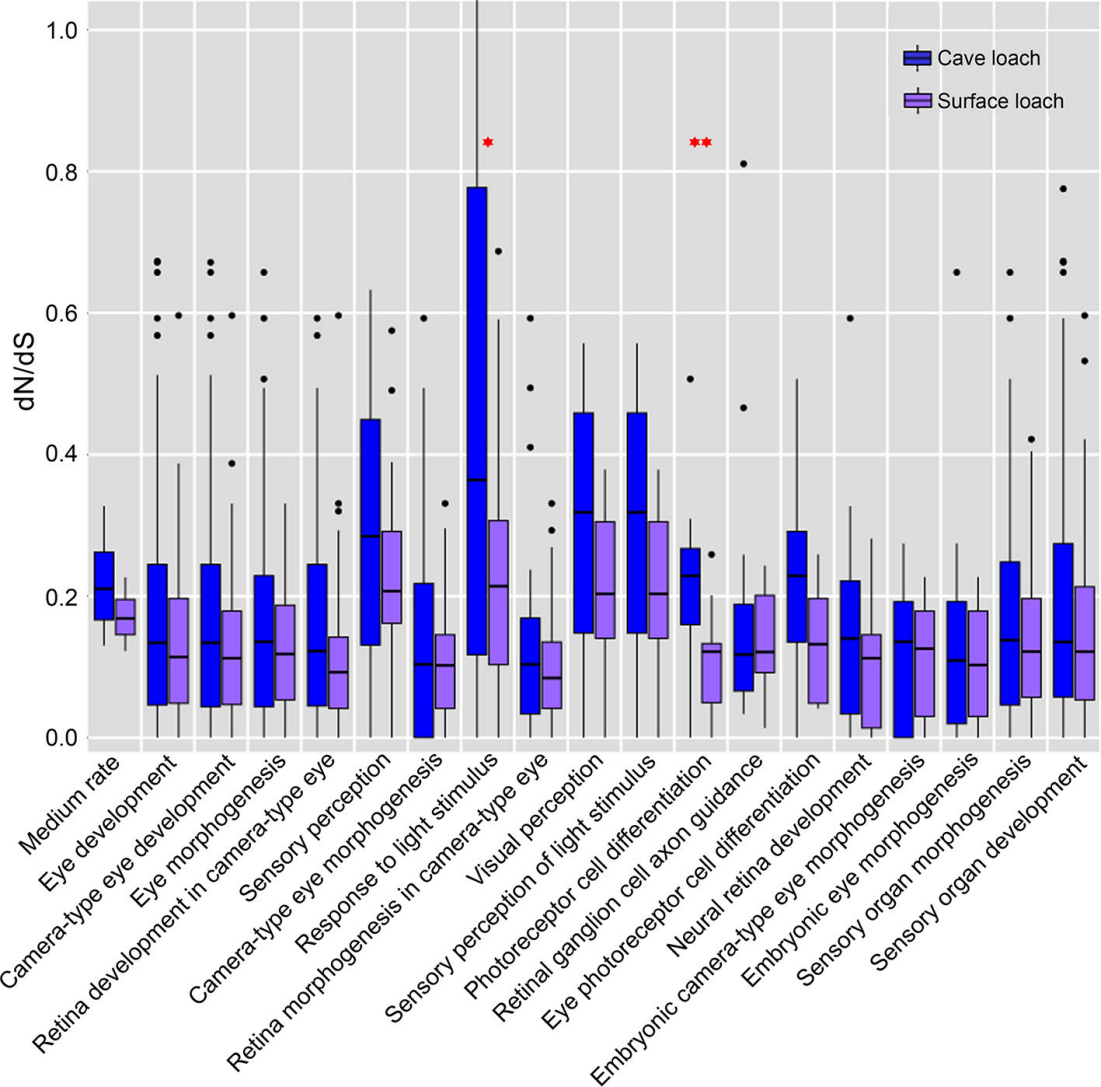
The dN/dS estimates in eye related gene-based GO functional categories. The analysis included all of the eye related GO categories of more than 10 genes. The genes of cave loach in the four directly visual related GO categories have higher dN/dS values. Specially, the GO categories of photoreceptor differentiation, cavefish have higher dN/dS than surface loach (^¬^P < 0.05; ^¬^^¬^P < 0.01; calculated by Two-tailed T test).

Further comparison and analysis revealed that in both species of loach, the four GO terms directly related to vision (“sensory perception”, “response to light stimulus”, “visual perception”, and “sensory perception of light stimulus”) had a higher dN/dS value (mean dN/dS value: 0.259, 0.552, 0.287, and 0.287 for *T. rosa*; 0.221, 0.204, 0.196, and 0.196 for *T. bleekeri*) than the medium rate (0.216 for *T. rosa* and 0.171 for *T. bleekeri*). Moreover, the cave loach had higher dN/dS values than the surface loach. In addition, we identified two GO terms, “photoreceptor cell differentiation” (0.213 for *T. rosa* and 0.103 for *T. bleekeri*) and “eye photoreceptor cell differentiation” (0.214 for *T. rosa* and 0.142 for *T. bleekeri*), for which the dN/dS value of the cave loach was higher than that of the surface loach, although these did not differ significantly from the medium rate bin values (**Figure 7**). These molecular results are consistent with the abnormal photoreceptor cell structure of the cave loach and suggest that during the process of dark adaptation in cavefish, vision-specific genes might be involved in visual degeneration and subjected to relaxed selection. Furthermore, we found that some vision-related genes had a higher evolutionary rate with respect to protein sequences (dN/dS > 1, FDR > 0.1, **Table 3**). Finally, we reasoned that it is not merely the dark environment that might promote the adaptive evolution of vision and that other light-dependent functional genes could also be involved. Moreover, we also identified several GO terms such as “photoperiodism”, “rhythmic process”, “response to UV”, and “DNA photolyase activity” that contained more genes in the high rate bin (**Table S6**).

**Table 3:** The vision-related genes which have a higher evolutional rate of protein sequence (high dN/dS rate).

Gene Symbol	Gene description	dN	dS	dN/dS
***rbp4l***	Retinol binding protein 4, like	0.004345	0.000004	1086.25
***cntfr***	Ciliary neurotrophic factor receptor	0.006323	0.000006	1053.833
***crybb1***	Crystallin beta b1	0.005243	0.000005	1048.6
***rhol***	Rhodopsin, like	0.017892	0.000018	994
rho*	Rhodopsin	0.003919	0.000004	979.75
***vsx2***	Visual system homeobox 2	0.005651	0.000006	941.8333
***nyx***	Nyctalopin	0.016646	0.000023	723.7391
***rx3***	Retinal homeobox gene 3	0.00207	0.000004	517.5
***grifin***	Galectin-related inter-fiber protein	0.009701	0.002692	3.60364
***rlbp1b***	Retinaldehyde binding protein 1b	0.009507	0.005155	1.844229
***opn7d***	Opsin 7, group member d	0.011402	0.008463	1.347276
***pde6c***	Phosphodiesterase 6c	0.008918	0.006839	1.303992
***rdh8a***	Retinol dehydrogenase 8a	0.004002	0.003144	1.272901

*downregulated gene.

Analysis of the relationship between gene expression and dN/dS revealed that the average gene expression level was negatively correlated with the dN/dS rate (slope: -0.0645; p: 7.585e-8; **Figure S3**). However, we found that the dN/dS values of upregulated genes in *T. rosa* eyes did not differ significantly from those of downregulated genes (**Figure S4**). Furthermore, we found that a differentially expressed vision-related gene (*rho*) had a higher evolutionary rate (dN/dS > 1, FDR > 0.1, **Table 3**). We did, however, identify six positively selected genes (**Table S5**) that were differentially expressed, although their function was not closely related to processes of interest in the present study.

### Premature Stop Codons and Frame-Shift Mutations in Vision-Related Genes

**Figure 8:**
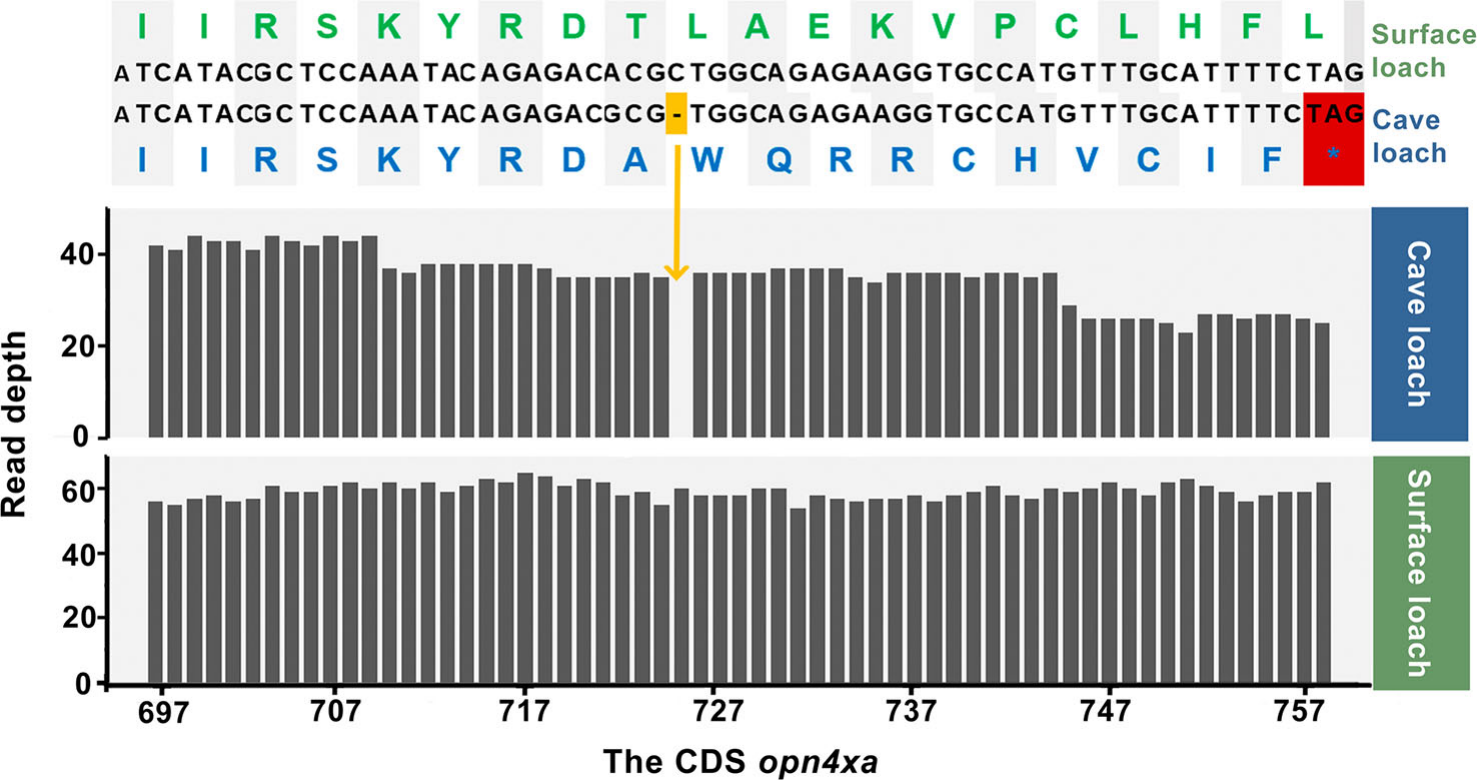
Alignment of nucleotide and amino acid sequences (top) and sequencing read depth (bottom; the numbers along the x-axis represent the position of the base at the CDS) for the *opn4xa* gene. The premature termination (colored red) of *opn4xa* is due to a single nucleotide insertion (colored orange).

We used genewise to predict the possible premature stop codons and frame-shift mutations in transcripts, and accordingly identified two genes related to light perception and vision. We detected a frame-shift mutation in *opn4xa* in cave loach, a light-sensitive protein associated with periodic rhythms, in which a base deletion had occurred at position 725 of the coding sequence (total length 1,401 bp), leading to the frame-shift in the middle section of the sequence. This mutation is assumed to have severely disrupted that the structure and function of the gene (**Figure 8**). On verifying the results obtained from the Bowtie alignments, we found that the gene was assembled correctly, which was consistent with the IGV result. Replacement of the missing base of *opn4xa* and subsequent Bowtie alignment confirmed that a base deletion had occurred in *opn4xa*. Given that we analyzed samples comprising the pooled genetic material of eight individuals, we are reasonably confident that this mutation might be present in most individuals of cave loaches. In addition, we identified another gene, *crybb3*, encoding an important part of the eye lens, in which 11 bases had been lost in cave loach (**Figure S5**), leading to a frame-shift mutation at the end of the protein, which was verified using the previously mentioned methods.

## Discussion

The results of our morphological analyses revealed that the lens and retinas of cave loach eyes are characterized by numerous deficiencies, reflecting their lack of use in the subterranean environment. In particular, the absence of the outer segment of photoreceptors indicates that visual perception of the cave loach might be significantly impaired. Additionally, our comparative transcriptome analysis revealed that, with respect to the comparison between *T. rosa* and *T. bleekeri*, there are a larger number of DEGs in the eyes than in the other tissues we examined (**Figure S2**). These observations might indicate that the morphological characteristics and the molecular mechanism underlying the formation of *T. rosa* eyes have been subjected to more changes in the process of adapting to the subterranean environment than the brain, gill, and skin. In addition, we found that the GO category “structural constituent of eye lens” was significantly enriched with genes shown to be downregulated in the eyes of cave loach. Further, enrichment analysis of the downregulated vision-associated genes from cavefish indicated that certain GO categories related to vision had low P-values (**Table 2**). These RNA-seq results were consistent with our morphological observations of abnormal lens and retina.

In *Astyanax,* the lens has been found to be fundamental in regulating eye degeneration, and in the present study, we detected the downregulation of the lens-related genes *pitx3*, *lim2*, and β|γ-crystallin in the *T. rosa* transcriptome. *pitx3* plays a critical role during lens development, with mutations of this gene being linked to anterior segment mesenchymal dysgenesis and congenital cataracts in humans (Sakazume et al., 2007), as well as the generation of small eyes lacking a lens in mice (Rieger et al., 2001). Similarly, in zebrafish, *pitx3* mutations result in small eyes and abnormalities in lens development (Shi et al., 2005). QTL and expression analyses of the *Astyanax* cavefish genome have revealed that *pitx3* might be related to eye degeneration (Mcgaugh et al., 2014). *Lim2* has been observed to localize to junctional regions of the lens fiber cell membrane, as well as being distributed throughout fiber cell membranes, suggesting a role in lens junctional communication, and mutations in this gene have also been associated with cataract formation (Tenbroek et al., 1992; Ponnam et al., 2008). β and γ-crystallin are members of the three main crystalline families associated with eye degeneration in cavefish, and previous studies in *Astyanax* have indicated that the lens plays a critical role in promoting cell survival during eye development (Strickler et al., 2007b). The abnormal functioning of these crystallin proteins can also lead to the development of cataracts (Yi et al., 2011). In particular, β- and γ-crystallins play a pivotal role in retinal tissue remodeling and repair, and strongly enhance axon regeneration in retinal ganglion cells (Thanos et al., 2014; Yang et al., 2016). These molecular studies confirm that the eyes of *T. rosa* are characterized by a degenerate lens and retina.

Teleosts have three *rx-* (*Rax-*) gene members, which are essential for organogenesis of the vertebrate eye. *Rx1* and *rx2* belong to the *Rax2* subgroup, with the former functioning in cell proliferation and differentiation during later development, while maintaining the identity and proliferative activity of retinal progenitors within the retina (Nelson et al., 2009; Orquera and De Souza, 2016). In zebrafish, depletion of *rx1* has been demonstrated to be lethal to retinal progenitors, which are necessary for rod and cone differentiation, as well as retinal neurogenesis (Nelson et al., 2009), whereas in *Astyanax* cavefish, *rx1* is expressed at low levels in the outer nuclear layer where photoreceptors are located (Strickler et al., 2002). Similarly, the expression of *rx1* has been found to be downregulated in the eyes of the cave loach. In contrast, *S. anophthalmus* and *S. anshuiensis* do not show differential expression of *rx1* (Meng et al., 2013; Yang et al., 2016). Although a perturbation of *rx2* function is not obviously manifested with respect to phenotypic changes (Chuang and Raymond, 2001; Nelson et al., 2009; Reinhardt et al., 2015), the expression of this gene is downregulated in *S. anophthalmus*, whereas it is normally expressed in *A. mexicanus* and *S. anshuiensis* (Meng et al., 2013; Li, 2016; Yang et al., 2016). The *Rax1* subgroup member *rx3* is the main gene involved in early eye structure development (Orquera and De Souza, 2016), and has been implicated in eye degeneration in *Astyanax* cavefish (Mcgaugh et al., 2014) but not in *Sinocyclocheilus*. *Crx* plays an important role in the differentiation and maintenance of photoreceptors and appears to be at the top of the retinal gene regulatory hierarchy (Meng et al., 2013; Homma et al., 2017; Assawachananont et al., 2018; Ruzycki et al., 2018). Mutations in this gene have been found to be associated with macular dystrophy (Griffith et al., 2018), photoreceptor defects (Ruzycki et al., 2017), and retinitis pigmentosa (Zhu et al., 2019), among other disorder. *Rx* activity does not regulate the expression of *crx* during photoreceptor development (Nelson et al., 2009), and *crx* downregulation in *S. anophthalmus* cavefish is a major contributor to the inactivity of retina-specific genes (with the exception of *rx1*) and small eyes (Meng et al., 2013). Changes in the expression of *crx* have also been implicated in the retinal degeneration of *Astyanax* and *Sinocyclocheilus* cavefish (Jeffery, 2009; Meng et al., 2013; Mcgaugh et al., 2014; Yang et al., 2016), and in the present study, we detected the downregulated expression of this gene and associated downstream transcription factors (e.g., *gnat2* and *nr2e3*). This decrease in expression might contribute to cave loach retinal degeneration and an anomalous photoreceptor layer.

The outer segment of photoreceptors, which contains numerous photosensitive and signal-transduced proteins, functions to transduce light signals into graded membrane potentials (Goldberg, 2006). The *prph2* (P/rds) gene encodes the core component of a multi-protein plasma membrane-rim-disc complex in the outer segment rim regions. This protein also contributes to the formation and daily renewal of outer segment disks in both rods and cones (Young, 1976; Molda et al., 1987; Boesze-Battaglia et al., 1998). In mice, *prph2* is essential for photoreceptor outer segment formation and its absence specifically abrogates outer segment development and causes a complete lack of outer segment organization, along with an increased number of photoreceptors characterized by retinal pigment epithelium (Sanyal and Jansen, 1981; Zulliger et al., 2018). Mutations in *prph2* are associated with autosomal-dominant retinitis pigmentosa and multiple classes of macular degeneration (Chakraborty et al., 2016). Furthermore, QTL analysis of *Astyanax* has revealed that *prph2a* might be associated with retinal degeneration (O'Quin et al., 2013) and transcriptome analysis has indicated that expression of this gene is reduced in adult cave *Astyanax* (Gross et al., 2013). In the present study, we found that the expression of *prph2* was markedly downregulated in *T. rosa*, which is consistent with the morphological evidence indicating a severely defective outer segment and lack of functionality in the photosensitive system. These results are also consistent with the observed downregulation of genes encoding visual proteins (*rho*, *pde6g*, and *rgrb*).

Studies on cave *Astyanax* and *Sinocyclocheilus* have to date yielded little evidence to suggest that changes in protein sequences might play a role in the visual degeneration of cavefish and have instead tended to indicate that the degeneration of cavefish eyes is mainly related to the regulation of gene expression or epigenetic inheritance (Hinaux et al., 2013; Mcgaugh et al., 2014; Hinaux et al., 2015; Casane and Rétaux, 2016; Yang et al., 2016; Gore et al., 2018). However, studies on the evolution of visual degeneration in cave mammals have indicated that this may not necessary hold true in all cases. Although the deterioration observed in the eyes of cave mammals is related to the regulation of gene expression, it is also inseparable from the accelerated evolution of a large number of vision-specific genes (Prudent et al., 2016; Partha et al., 2017). In this regard, the findings of the present study indicate that the differences between cave mammals and cavefish are probably associated with the differential evolutionary rates of the respective nucleic acids. Cavefish are confined to water-containing caves and the limited food resources therein require them to slow their metabolic rate to survive through long periods of starvation (Hervant et al., 1998; Hervant et al., 1999; Hervant et al., 2001; Shi et al., 2018). Moreover, the low metabolic rate is associated with a low rate of nucleic acid evolution (Martin and Palumbi, 1993). As such, this may have made it difficult to detect changes in the sequences of proteins that are also involved in visual degradation.

In the present, however, we obtained higher dN replacement rate and high dN/dS values for cave loach than for the surface loach, suggesting that changes in protein sequences are indeed involved in the process whereby the cave loach adapts to the cave environment. Given that we have previously excluded the possibility of the effect small population size (Zhao et al., 2014; Xue et al., 2015), we suspect that the observed high dN rates could be due to factors such as positive or relaxed selection (Comeron and Kreitman, 1998; Subramanian, 2013). However, we detected no positively selected genes directly related to vision in the present study. Accordingly, it is conceivable that changes in protein sequences might be involved in adaptation of the cave loach and that the degeneration of eyes might be associated with relaxed selection.

In an effort to gain a better understanding the evolutionary status of eye-related genes, we divided the genes into three bins according to dN/dS values and subsequently performed GO enrichment analysis on these three types of genes. Although the same gene can be classified into multiple GO categories, the trend of GO clustering might indicate that genes directly related to vision have higher dN/dS values than genes associated with eye development. In this regard, we identified four GO terms in cave loach directly related to vision that had higher dN/dS values than the dN/dS values of the medium rate bin and the surface loach. Moreover, compared with the surface loach, we conjecture that the evolutionary rate of photoreceptor differentiation-related genes in the cave loach may be higher than that in the surface loach. Indeed, the results of coding sequence analysis indicated that the proteins directly related to vision are likely to have a higher rate of evolution and might be involved in cave loach eye degeneration, which is consistent with our morphological results showing evidence of the degeneration of the retina and the absence of the outer segment of photoreceptor cells. In contrast, the genes associated with eye development appear to be more conserved, which might be related to the fact that these genes are not vision-specific genes (Partha et al., 2017). Accordingly, these findings may indicate that although cavefish and cave mammals have evolutionary similar degenerative mechanisms, the respective rates of the evolutionary processes may differ at the molecular level.

Further, there are some genes associated with vision that have a high rate of protein evolution and thus might be candidate genes involved in eye degeneration in the cave loach (**Table 3**). We detected large changes in the amino acid sequence of three genes that might be involved in degeneration of the lens, namely, a frame-shift mutation in *crybb3* and higher evolutionary rates of *crybb1* and a griffin protein (galectin-related inter-fiber protein) (**Table 3**). *Crybb1* and *crybb3* belong to the β|γ-crystallin family and play important roles in maintaining the normal structure of the lens. Moreover, griffin, a lens-specific protein related to the galectin family in mammals, birds, and early embryos of zebrafish, interacts with crystallin and plays an important role in development of the lens (Ahmed and Vasta, 2008; Barton et al., 2009; Caballero et al., 2019). These observations may thus indicate that *T. rosa* has a degenerate lens.

Visual system homeobox 2 (*vsx2*) is a key transcription factor involved in neural retinal development (Sigulinsky et al., 2015), and mutations in this gene can cause microphthalmia (Ammar et al., 2017). The encoded protein sequence has a relatively high evolutionary rate in cavefish, indicating that the abnormal morphology and small eyes of the cavefish might also be associated with changes in this gene. *Rx3* also has a high protein evolution rate in cave loach, with a dN/dS value considerably higher than 1, thereby indicating that this gene might also be involved in the degeneration of the cave loach retina. Furthermore, the high rate of evolution or rapid evolution of genes (**Table 3**) related to visual perception might also be associated with eye degeneration and are consistent with the degradation of photosensitivity and the absence of the outer segment in cave loach photoreceptor cells.

Finally, we found that some genes related to non-visual photoreceptors also had altered protein sequences. *Opn4xa* is one of five melanopsins in zebrafish and plays a role in non-image-forming light functions, including the photoentrainment of circadian rhythms (Davies et al., 2011; Matos-Cruz et al., 2011). The frame-shift mutation in *opn4xa* and the higher evolutionary rate of photoperiod-related genes also indicate that photoperiod regulation in cavefish might be altered. In addition, genes related to UV damage repair also exhibit a higher rate of protein evolution, indicating that UV damage repair might not be important in a dark environment.

## Conclusions

In this study we compared eye morphology and transcriptome sequence divergence between *T. rosa* and *T. bleekeri*, and thereby confirmed that eye degeneration is a distinct troglomorphic characteristic of the former. The cavefish has reduced eye size, anomalous lens morphology, retinal pyknotic nuclei, and photoreceptor deficiencies. At the molecular level, lens degeneration was found to be associated with the transcription factor *pitx3*, which controls lens development, and downregulation of intrinsic lens proteins (β- and γ-crystallins). Additionally, we demonstrate that retinal defects might be linked to the transcription factor *crx* and its downstream transcription factor genes that are involved in retinal development, vision-related genes, and the downregulation of structural protein genes of the photoreceptor outer segment. We also found that changes in protein sequence were consistent with the production of degenerate eye phenotypes. Importantly, we provide evidence indicating that changes in protein sequences might be involved in visual degeneration. This is the first time that we have identified vision-specific genes in cavefish with a higher rate of evolution at the protein level, and which might be involved in the degenerative evolution of vision. Our findings also reveal that the evolutionary trend of vision-specific genes might be associated with relaxed selection, characterized by the gradual and slow accumulation of mutations. Moreover, we identified a number of vision-related genes that are commonly downregulated in *T. rosa*, *Sinocyclocheilus*, and *Astyanax* cavefish, some of which (*rax-*, β|γ-crystallin, *hsp90a*) might exhibit differential expression patterns (**Table S7**). Collectively, these finding have provided important insights regarding the diverse mechanisms underlying cavefish eye degeneration and suggest that even similar cavefish phenotypes might be the result of different processes or different mutations. However, the transcriptome does not provide all the genetic information of a species and our study is also limited with respect to the fact that we only examined adult organisms. A complete explanation of the degenerative mechanisms of the eye in *T. rosa*, or in cavefish generally, requires further investigation of the genomes, transcriptional regulation, pseudogenization, and developmental biology of these fish.

## Data Availability Statement

The raw data has been deposited in Sequence Read Archive of National Center for Biotechnology Information database (NCBI-SRA) and under BioProject PRJNA418202.

## Ethics Statement

The animal study was reviewed and approved by The Animal Care and Use Committee of Southwest University.

## Author Contributions

Project administration and conceptualization: ZP. Sampling and RNA extraction: YL and YX. Histology: YN. Data analysis and writing: QZ and FS. Expression verification: YX. Article review: ZP and RZ. All authors discussed results and commented on the manuscript.

## Funding

This work was supported by the grants from the National Natural Science Foundation of China (31572254 and 31872204).

## Conflict of Interest

The authors declare that the research was conducted in the absence of any commercial or financial relationships that could be construed as a potential conflict of interest.
