# Supplementary material for: Comparative Transcriptome Profiling of the Loaches Triplophysa bleekeri and Triplophysa rosa Reveals Potential Mechanisms of Eye Degeneration
Source: Front Genet. 2020 Jan 16;10:1334. doi: 10.3389/fgene.2019.01334 (PMC6977438; doi:10.3389/fgene.2019.01334)
Supplement: Supplementary file 1 [file DataSheet_1.docx]

**Table S1.** Primers used for the qRT-PCR analysis of genes in the *Trilophysa* transcriptomes.

| Gene ID | Description | Forward primer (5′–3′) | Reverse primer (5′–3′) | Tissue |
| --- | --- | --- | --- | --- |
| *tufm* | Tu translation elongation factor, mitochondrial | GGAAGTTCAGGCAGCAGTG | TAGTGGTATGGAGAAGATTGTAGG | eyes |
| *crybb3* | Crystallin beta B3 | ACCAACAGTCAGAACAGC | CATCATCGTCCACAATTTCC | eyes |
| *cryba1* | Crystallin beta A1 | CCTCAGCCTGTCAGAACG | ATTAGCCTCTCAATGTGATATGC | eyes |
| *crygm3* | Crystallin gamma M3 | CTGATGACTGCGACTCTG | TGTATCCCATATCCCTAAAGC | eyes |
| *hspb1* | Heat shock protein family B (small) member 1 | CTACCTCACCACCTCTAATG | GAAGTCCTCTGTCTCTGC | eyes |
| *hprt1* | Hypoxanthine phosphoribosyltransferase 1 | TGCCAGCCCTTGTGTCGTG | TCGGTTCTGTCCATGATGAGTCC | eyes |
| *actb* | Actin beta | CAGCAGATGTGGATTAGCAAGCAG | TGAGTCGGCGTGAAGTGGTAAC | eyes |
| *rx1* | Retinal homeobox protein | CTGCCAGAGGTCCGAGTGC | GAGGTCAGCCAAGGGTCAAGG | eyes |
| *gnat2* | G Protein subunit alpha transducin 2 | TTCGGGTCACCAGCATCACAG | ACGCCTGAATCCTTCCATAGCC | eyes |
| *cacna1d* | Calcium voltage-gated channel subunit alpha1 D | ACGCTGTTACGCCGCATTC | GTCCCGTCGCTGTTGAGAGG | eyes |
| *rorb* | RAR related orphan receptor B | TGGCAGACGCACTCCTACG | ACCAGCACAACTTCCAAACAACC | eyes |
| *lim2* | Lens intrinsic membrane protein 2 | TCCACGGCGACGGACTATTG | CATTCCAGTATGCTTGAACGAACC | eyes |
| *opn1sw* | Opsin 1, short wave sensitive | CCGCCTGTGGACCTGACTG | ACACTTCCTTCTCTGCCTTCTGG | eyes |
| *hspb2* | Heat shock protein family B (small) member 2 | ACATCCGTCCCCGCATCAAC | ACCGTGCTCGTCCATTCTCTG | eyes |
| *rps2* | Ribosomal protein S2 | AGCCTCTTCTAACGGGAATGATGG | GCCTTTGTTGCTATCGGTGACTAC | eyes |
| *crx* | Cone-rod homeobox | GCCAACAACAACAGCAATCCAGTG | GTGCCAGCATCAGAGGTCAGC | eyes |
| *rgrb* | Retinal G protein coupled receptor b | TGGATCGGAGGGATGTCAGTTG | TCATAGGCAGCGGTAGAGCAG | eyes |
| *prph2* | Peripherin 2 (retinal degeneration, slow) | GATGTTAAGGTCCGTGTTCAGAGC | TGTTGGTTATGTGATGCTGGATGC | eyes |
| *pitx3* | Paired like homeodomain 3 | GCCCACACCAACAAACTCAAACC | TCACAAGCGAGACTGTAGCATCC | eyes |
| *svil* | Supervillin | GGACGAGGTTATGGATTGGTGGAG | TGCCGACTGCTGTGCTGAC | eyes |
| *rho* | Rhodopsin | TCGGCGGATTCACCACAACG | GCACAACCAGGGACCAGAGC | eyes |
| *pde6g* | Phosphodiesterase 6G | TGCCCAGATTTTATTGGTTGTTCG | ATCTCCTTCGCTCTGTCTTCCTC | eyes |

**Table S2.** The integrity assessment from CEGMA (based on 248 core genes).

| Species | Completeness % | Partial % |
| --- | --- | --- |
| *T. rosa* | 98.79 | 99.60 |
| *T. bleekeri* | 99.60 | 100 |

**Table S3.** 21 GO categories were significantly enriched in up regulated genes of cave loaches.

| **GO ID** | **Description** | **GeneRatio** | **BgRatio** | **pvalue** | **fdr** |  |
| --- | --- | --- | --- | --- | --- | --- |
| GO:0005576 | Extracellular region | 187 | 1836 | 1.28E-05 | 0.0070 | Celluler Component |
| GO:0005615 | Extracellular space | 51 | 391 | 0.00011 | 0.031 | Celluler Component |
| GO:0042611 | MHC protein complex | 10 | 38 | 0.00042 | 0.077 | Celluler Component |
| GO:0016494 | C-X-C chemokine receptor activity | 5 | 5 | 2.04E-06 | 0.0021 | Molecular Function |
| GO:0001637 | G-protein coupled chemoattractant receptor activity | 6 | 9 | 1.03E-05 | 0.0036 | Molecular Function |
| GO:0004950 | Chemokine receptor activity | 6 | 9 | 1.03E-05 | 0.0036 | Molecular Function |
| GO:0004866 | Endopeptidase inhibitor activity | 28 | 165 | 2.25E-05 | 0.0041 | Molecular Function |
| GO:0030414 | Peptidase inhibitor activity | 31 | 193 | 2.58E-05 | 0.0041 | Molecular Function |
| GO:0061135 | Endopeptidase regulator activity | 28 | 167 | 2.82E-05 | 0.0041 | Molecular Function |
| GO:0004857 | Enzyme inhibitor activity | 42 | 299 | 3.13E-05 | 0.0041 | Molecular Function |
| GO:0061134 | Peptidase regulator activity | 31 | 195 | 3.17E-05 | 0.0041 | Molecular Function |
| GO:0008289 | Lipid binding | 60 | 487 | 4.32E-05 | 0.0050 | Molecular Function |
| GO:0004198 | Calcium-dependent cysteine-type endopeptidase activity | 10 | 34 | 0.00011 | 0.011 | Molecular Function |
| GO:0030246 | Carbohydrate binding | 34 | 266 | 0.00098 | 0.093 | Molecular Function |
| GO:0004052 | Arachidonate 12-lipoxygenase activity | 3 | 4 | 0.0015 | 0.095 | Molecular Function |
| GO:0016165 | Linoleate 13S-lipoxygenase activity | 3 | 4 | 0.0015 | 0.095 | Molecular Function |
| GO:0070915 | Lysophosphatidic acid receptor activity | 3 | 4 | 0.0015 | 0.095 | Molecular Function |
| GO:0003690 | Double-stranded DNA binding | 21 | 142 | 0.0015 | 0.095 | Molecular Function |
| GO:0001608 | G-protein coupled nucleotide receptor activity | 4 | 8 | 0.0015 | 0.095 | Molecular Function |
| GO:0045028 | G-protein coupled purinergic nucleotide receptor activity | 4 | 8 | 0.0015 | 0.095 | Molecular Function |
| GO:0002376 | Immune system process | 63 | 502 | 1.44E-05 | 0.046 | Biological Process |

**Table** **S4.** The GO categories that were significantly enriched involved in eye down DEGs.

| **GO ID** | **Description** | **GeneRatio** | **BgRatio** | **pvalue** | **fdr** |  |
| --- | --- | --- | --- | --- | --- | --- |
| GO:0016020 | Membrane | 500 | 7794 | 4.41E-10 | 2.06E-07 | Celluler Component |
| GO:0031224 | Intrinsic component of membrane | 300 | 4496 | 2.01E-06 | 0.00034 | Celluler Component |
| GO:0016021 | Integral component of membrane | 297 | 4447 | 2.18E-06 | 0.00034 | Celluler Component |
| GO:0044425 | Membrane part | 348 | 5420 | 7.44E-06 | 0.00087 | Celluler Component |
| GO:1902495 | Transmembrane transporter complex | 28 | 221 | 1.97E-05 | 0.0017 | Celluler Component |
| GO:1990351 | Transporter complex | 28 | 222 | 2.14E-05 | 0.0017 | Celluler Component |
| GO:0034702 | Ion channel complex | 22 | 180 | 0.00025 | 0.017 | Celluler Component |
| GO:0045202 | Synapse | 20 | 159 | 0.00032 | 0.019 | Celluler Component |
| GO:0044456 | Synapse part | 16 | 115 | 0.0004 | 0.021 | Celluler Component |
| GO:0044459 | Plasma membrane part | 61 | 743 | 0.00054 | 0.025 | Celluler Component |
| GO:0005578 | Proteinaceous extracellular matrix | 36 | 379 | 0.00061 | 0.026 | Celluler Component |
| GO:0034703 | Cation channel complex | 19 | 157 | 0.00073 | 0.026 | Celluler Component |
| GO:0008076 | Voltage-gated potassium channel complex | 13 | 88 | 0.00078 | 0.026 | Celluler Component |
| GO:0034705 | Potassium channel complex | 13 | 88 | 0.00078 | 0.026 | Celluler Component |
| GO:0030054 | Cell junction | 25 | 239 | 0.0011 | 0.033 | Celluler Component |
| GO:0098797 | Plasma membrane protein complex | 41 | 465 | 0.0012 | 0.033 | Celluler Component |
| GO:0045211 | Postsynaptic membrane | 10 | 60 | 0.0012 | 0.033 | Celluler Component |
| GO:0031012 | Extracellular matrix | 40 | 458 | 0.0016 | 0.041 | Celluler Component |
| GO:0097060 | Synaptic membrane | 10 | 64 | 0.002 | 0.049 | Celluler Component |
| GO:0031226 | Intrinsic component of plasma membrane | 32 | 350 | 0.0022 | 0.051 | Celluler Component |
| GO:0005886 | Plasma membrane | 91 | 1275 | 0.0026 | 0.057 | Celluler Component |
| GO:0098796 | Membrane protein complex | 101 | 1462 | 0.0041 | 0.087 | Celluler Component |
| GO:0098794 | Postsynapse | 10 | 72 | 0.0048 | 0.096 | Celluler Component |
| GO:0005861 | Troponin complex | 5 | 22 | 0.0053 | 0.096 | Celluler Component |
| GO:0005865 | Striated muscle thin filament | 5 | 22 | 0.0053 | 0.096 | Celluler Component |
| GO:0036379 | Myofilament | 5 | 22 | 0.0053 | 0.096 | Celluler Component |
| GO:0005212 | Structural constituent of eye lens | 7 | 11 | 3.80E-07 | 0.00035 | Molecular Function |
| GO:0022836 | Gated channel activity | 43 | 399 | 1.59E-05 | 0.0059 | Molecular Function |
| GO:0022857 | Transmembrane transporter activity | 155 | 2068 | 1.93E-05 | 0.0059 | Molecular Function |
| GO:0015267 | Channel activity | 62 | 671 | 3.29E-05 | 0.006 | Molecular Function |
| GO:0022803 | Passive transmembrane transporter activity | 62 | 671 | 3.29E-05 | 0.006 | Molecular Function |
| GO:0019905 | Syntaxin binding | 7 | 21 | 8.26E-05 | 0.011 | Molecular Function |
| GO:0005215 | Transporter activity | 170 | 2371 | 8.28E-05 | 0.011 | Molecular Function |
| GO:0022838 | Substrate-specific channel activity | 59 | 653 | 9.48E-05 | 0.011 | Molecular Function |
| GO:0022891 | Substrate-specific transmembrane transporter activity | 138 | 1870 | 0.00012 | 0.012 | Molecular Function |
| GO:0005216 | Ion channel activity | 58 | 648 | 0.00014 | 0.013 | Molecular Function |
| GO:0000149 | SNARE binding | 7 | 23 | 0.00016 | 0.013 | Molecular Function |
| GO:0005515 | Protein binding | 439 | 7070 | 0.00023 | 0.016 | Molecular Function |
| GO:0005509 | Calcium ion binding | 70 | 840 | 0.00026 | 0.016 | Molecular Function |
| GO:0005244 | Voltage-gated ion channel activity | 22 | 177 | 0.00026 | 0.016 | Molecular Function |
| GO:0015075 | Ion transmembrane transporter activity | 130 | 1774 | 0.00026 | 0.016 | Molecular Function |
| GO:0022832 | Voltage-gated channel activity | 22 | 178 | 0.00028 | 0.016 | Molecular Function |
| GO:0022843 | Voltage-gated cation channel activity | 19 | 147 | 0.0004 | 0.022 | Molecular Function |
| GO:0022892 | Substrate-specific transporter activity | 142 | 2006 | 0.0006 | 0.03 | Molecular Function |
| GO:0005249 | Voltage-gated potassium channel activity | 13 | 87 | 0.00083 | 0.04 | Molecular Function |
| GO:0008509 | Anion transmembrane transporter activity | 23 | 206 | 0.00088 | 0.04 | Molecular Function |
| GO:0005230 | Extracellular ligand-gated ion channel activity | 16 | 126 | 0.0014 | 0.057 | Molecular Function |
| GO:0015276 | Ligand-gated ion channel activity | 23 | 214 | 0.0015 | 0.057 | Molecular Function |
| GO:0022834 | Ligand-gated channel activity | 23 | 214 | 0.0015 | 0.057 | Molecular Function |
| GO:0015103 | Inorganic anion transmembrane transporter activity | 11 | 71 | 0.0015 | 0.057 | Molecular Function |
| GO:0005261 | Cation channel activity | 31 | 326 | 0.0019 | 0.069 | Molecular Function |
| GO:0098772 | Molecular function regulator | 76 | 1001 | 0.002 | 0.072 | Molecular Function |
| GO:0098609 | Cell-cell adhesion | 40 | 245 | 2.89E-10 | 7.05E-07 | Biological Process |
| GO:0007155 | Cell adhesion | 75 | 704 | 1.02E-08 | 8.56E-06 | Biological Process |
| GO:0022610 | Biological adhesion | 77 | 731 | 1.05E-08 | 8.56E-06 | Biological Process |
| GO:0007268 | Synaptic transmission | 26 | 163 | 5.98E-07 | 0.00022 | Biological Process |
| GO:0099536 | Synaptic signaling | 26 | 163 | 5.98E-07 | 0.00022 | Biological Process |
| GO:0099537 | Trans-synaptic signaling | 26 | 163 | 5.98E-07 | 0.00022 | Biological Process |
| GO:0007156 | Homophilic cell adhesion via plasma membrane adhesion molecules | 27 | 174 | 6.41E-07 | 0.00022 | Biological Process |
| GO:0007267 | Cell-cell signaling | 29 | 204 | 1.60E-06 | 0.00048 | Biological Process |
| GO:0098742 | Cell-cell adhesion via plasma-membrane adhesion molecules | 27 | 183 | 1.76E-06 | 0.00048 | Biological Process |
| GO:0055085 | Transmembrane transport | 115 | 1445 | 1.11E-05 | 0.0027 | Biological Process |
| GO:0044699 | Single-organism process | 695 | 11857 | 1.44E-05 | 0.0032 | Biological Process |
| GO:0044765 | Single-organism transport | 229 | 3345 | 2.73E-05 | 0.0056 | Biological Process |
| GO:0016337 | Single organismal cell-cell adhesion | 13 | 65 | 3.51E-05 | 0.0066 | Biological Process |
| GO:0098602 | Single organism cell adhesion | 13 | 68 | 5.77E-05 | 0.01 | Biological Process |
| GO:1902578 | Single-organism localization | 231 | 3430 | 7.41E-05 | 0.012 | Biological Process |
| GO:0044763 | Single-organism cellular process | 585 | 9960 | 0.00041 | 0.063 | Biological Process |

**Table S5.** The genes were under positive selection in cave loach.

| **Gene symbol** | **Gene description** | **dN/dS** | **FDR** | **P-Value** |
| --- | --- | --- | --- | --- |
| ***plpp3*** | Phospholipid phosphatase 3 | 96.14 | 0 | 0.00E+00 |
| ***dip2bb*** | Disco-interacting protein 2 homolog Bb | 0.91 | 0 | 0.00E+00 |
| ***fam184a*** | Family with sequence similarity 184 member A | 11.68 | 4.68E-09 | 4.44E-11 |
| ***clcn3*^🟋^** | Chloride voltage-gated channel 3 | 6.55 | 1.47E-05 | 1.86E-07 |
| ***si:ch211-76l23.4*^#^** | The unknown protein | 30 | 6.72E-05 | 1.06E-06 |
| ***ttc9*** | Tetratricopeptide Repeat Domain 9 | 23.26 | 0.00021 | 3.96E-06 |
| ***acp1*** | Acid phosphatase 1 | 1.99 | 0.00031 | 6.88E-06 |
| ***prelid3a*** | PRELI domain containing 3A | 13.54 | 0.00033 | 1.11E-05 |
| ***cfdp1*** | Craniofacial Development Protein 1 | 6.29 | 0.00033 | 1.14E-05 |
| ***gnao1a*** | Guanine nucleotide binding protein (G protein), alpha activating activity polypeptide O, a | 3.61 | 0.00033 | 1.15E-05 |
| ***zgc:172067*^🟋^** | Uncharacterized protein LOC100006042 | 14.03 | 0.00033 | 1.15E-05 |
| ***dennd4a*** | DENN Domain Containing 4A | 3.62 | 0.00033 | 1.24E-05 |
| ***nab1b*** | NGFI-A binding protein 1 b | 18.37 | 0.0004 | 1.65E-05 |
| ***hps6*** | Biogenesis of lysosomal organelles complex 2 subunit 3 | 1.18 | 0.0022 | 1.02E-04 |
| ***lpar2b*^#^** | Lysophosphatidic acid receptor 2 b | 1.38 | 0.0022 | 1.05E-04 |
| ***uso1*** | Vesicle transport factor | 2.33 | 0.0032 | 1.62E-04 |
| ***xrcc2*** | X-ray repair cross complementing 2 | 8.15 | 0.005 | 2.70E-04 |
| ***col4a2*** | Collagen type IV alpha 2 chain | 1.47 | 0.0074 | 4.22E-04 |
| ***pou2f3*^#^** | POU class 2 homeobox 3 | 0.94 | 0.0082 | 4.93E-04 |
| ***txndc17*** | TXNDC17 promotes paclitaxel resistance via inducing autophagy in ovarian cancer. | 11.59 | 0.017 | 1.10E-03 |
| ***fxr2*** | FMR1 autosomal homolog 2 | 4.45 | 0.021 | 1.40E-03 |
| ***fam26f*** | Family with sequence similarity 26 member F | 3.89 | 0.023 | 1.58E-03 |
| ***cpxm1a*** | Carboxypeptidase X (M14 family), member 1a | 1.4 | 0.027 | 1.96E-03 |
| ***rnf24*** | Ring finger protein 24 | 9.13 | 0.031 | 2.43E-03 |
| ***lactb*** | Lactamase beta | 2.55 | 0.031 | 2.47E-03 |
| ***spryd3*** | SPRY domain containing 3 | 7.7 | 0.039 | 3.22E-03 |
| ***zgc:101723*** | Trans-1,2-dihydrobenzene-1,2-diol dehydrogenase | 2.4 | 0.041 | 3.54E-03 |
| ***si:ch211-106k21.5*** | Toll-like receptor 8 | 1.043 | 0.05 | 4.39E-03 |
| ***slc16a4*** | Solute Carrier Family 16 Member 4 | 1.025 | 0.05 | 4.59E-03 |
| ***atp1b3b*** | ATPase Na+/K+ Transporting Subunit Beta 3 | 1.52 | 0.058 | 5.51E-03 |
| ***p2ry4*** | Pyrimidinergic receptor P2Y4 | 4.36 | 0.061 | 6.03E-03 |
| ***ndnl2*** | Necdin-like 2 | 13.34 | 0.066 | 6.70E-03 |
| ***fnta*** | Farnesyltransferase, CAAX Box, Alpha | 1.54 | 0.067 | 7.05E-03 |
| ***zgc:153990*** | Uncharacterized protein LOC768125 | 4.01 | 0.073 | 7.97E-03 |
| ***rem1*** | RRAD And GEM Like GTPase 1 | 29.73 | 0.073 | 8.05E-03 |
| ***si:ch211-232b12.5*** | Uncharacterized protein si:ch211-232b12.5 | 1.15 | 0.073 | 8.56E-03 |
| ***scn4ba*** | Sodium channel, voltage-gated, type IV, beta a | 75.17 | 0.073 | 8.93E-03 |
| ***zfand5a*** | Zinc Finger AN1-Type Containing 5 | 26.43 | 0.073 | 9.05E-03 |
| ***mfap5*** | Microfibril associated protein 5 | 20.09 | 0.073 | 9.12E-03 |
| ***otud1*** | OTU Deubiquitinase 1 | 47.4 | 0.073 | 9.24E-03 |
| ***slc38a2*** | Solute carrier family 38 member 2 | 5.27 | 0.074 | 9.56E-03 |
| ***rragcb*** | Ras-related GTP binding Cb | 10.19 | 0.074 | 9.82E-03 |
| ***zgc:112332*** | Uncharacterized protein LOC553712 | 1.57 | 0.077 | 1.04E-02 |
| ***cebpa*** | CCAAT Enhancer Binding Protein Alpha | 1.96 | 0.08 | 1.12E-02 |
| ***serpinb1*** | Serpin family B member 1 | 2.67 | 0.082 | 1.18E-02 |
| ***phpt1*** | Phosphohistidine Phosphatase 1 | 3.53 | 0.082 | 1.20E-02 |
| ***itm2ba*** | Integral membrane protein 2Ba | 1.86 | 0.088 | 1.34E-02 |
| ***st3gal2*** | ST3 beta-galactoside alpha-2,3-sialyltransferase 2 | 54.96 | 0.088 | 1.38E-02 |
| ***zgc:92791*^#^** | Proteasome subunit beta 10 | 2.47 | 0.088 | 1.39E-02 |
| ***entpd5a*** | Ectonucleoside triphosphate diphosphohydrolase 5a | 2.01 | 0.088 | 1.39E-02 |
| ***cpox*** | Coproporphyrinogen oxidase | 1.64 | 0.091 | 1.47E-02 |

**Note:** **^🟋^**downregulated genes; **^#^**upregulated genes.

| **Table S6.** Visually related GO categories in GO enrichment analysis of three bins (according to dN/dS). | | | | | | | | | | | |
| --- | --- | --- | --- | --- | --- | --- | --- | --- | --- | --- | --- |
| **GO_ID** | **Description** | **High rate** | | | **Medium rate** | | | **Low rate** | | | **Total genes** |
|  |  | **Genes** | **P value** | **FDR** | **Genes** | **P value** | **FDR** | **Genes** | **P value** | **FDR** |  |
| GO:0090596 | Sensory organ morphogenesis | 15 | 0.99 | 1 | 25 | 0.55 | 1 | 34 | 0.032 | 0.43 | 74 |
| GO:0071482 | Cellular response to light stimulus | 2 | 0.1 | 1 |  |  |  |  |  |  | 2 |
| GO:0070654 | Sensory epithelium regeneration | 2 | 0.38 | 1 |  |  |  | 2 | 0.42 | 0.78 | 4 |
| GO:0061386 | Closure of optic fissure |  |  |  | 1 | 0.8 | 1 | 3 | 0.12 | 0.64 | 4 |
| GO:0061299 | Retina vasculature morphogenesis in camera-type eye | 1 | 0.32 | 1 |  |  |  |  |  |  | 1 |
| GO:0061298 | Retina vasculature development in camera-type eye | 1 | 0.32 | 1 |  |  |  |  |  |  | 1 |
| GO:0061072 | Iris morphogenesis |  |  |  | 1 | 0.33 | 0.98 |  |  |  | 1 |
| GO:0060898 | Eye field cell fate commitment involved in camera-type eye formation |  |  |  |  |  |  | 1 | 0.34 | 0.71 | 1 |
| GO:0060219 | Camera-type eye photoreceptor cell differentiation |  |  |  | 2 | 0.25 | 0.98 | 1 | 0.71 | 0.87 | 3 |
| GO:0060059 | Embryonic retina morphogenesis in camera-type eye | 1 | 0.93 | 1 |  |  |  | 6 | 0.0076 | 0.18 | 7 |
| GO:0060042 | Retina morphogenesis in camera-type eye | 4 | 0.95 | 1 | 6 | 0.78 | 1 | 12 | 0.037 | 0.46 | 22 |
| GO:0060041 | Retina development in camera-type eye | 7 | 0.99 | 1 | 16 | 0.65 | 1 | 27 | 0.0039 | 0.12 | 50 |
| GO:0051786 | All-trans-retinol 13,14-reductase activity |  |  |  | 1 | 0.11 | 0.5 |  |  |  | 1 |
| GO:0050962 | Detection of light stimulus involved in sensory perception | 1 | 0.68 | 1 | 2 | 0.25 | 0.98 |  |  |  | 3 |
| GO:0050953 | Sensory perception of light stimulus | 8 | 0.18 | 1 | 8 | 0.21 | 0.98 | 2 | 0.99 | 1.00 | 18 |
| GO:0050908 | Detection of light stimulus involved in visual perception | 1 | 0.68 | 1 | 2 | 0.25 | 0.98 |  |  |  | 3 |
| GO:0050906 | Detection of stimulus involved in sensory perception | 3 | 0.58 | 1 | 5 | 0.14 | 0.98 | 1 | 0.98 | 1.00 | 9 |
| GO:0048880 | Sensory system development | 8 | 0.46 | 1 | 5 | 0.92 | 1 | 9 | 0.37 | 0.75 | 22 |
| GO:0048596 | Embryonic camera-type eye morphogenesis | 1 | 0.98 | 1 | 5 | 0.28 | 0.98 | 5 | 0.3 | 0.71 | 11 |
| GO:0048593 | Camera-type eye morphogenesis | 5 | 0.99 | 1 | 12 | 0.55 | 1 | 19 | 0.015 | 0.28 | 36 |
| GO:0048592 | Eye morphogenesis | 8 | 0.99 | 1 | 19 | 0.34 | 0.99 | 24 | 0.045 | 0.49 | 51 |
| GO:0048511 | Rhythmic process | 3 | 0.49 | 1 | 4 | 0.25 | 0.98 |  |  |  | 7 |
| GO:0048069 | Eye pigmentation |  |  |  |  |  |  | 1 | 0.34 | 0.71 | 1 |
| GO:0048048 | Embryonic eye morphogenesis | 2 | 0.99 | 1 | 6 | 0.51 | 1 | 9 | 0.083 | 0.64 | 17 |
| GO:0046549 | Retinal cone cell development |  |  |  |  |  |  | 1 | 0.34 | 0.71 | 1 |
| GO:0046530 | Photoreceptor cell differentiation | 2 | 0.98 | 1 | 11 | 0.0016 | 0.91 | 2 | 0.98 | 1.00 | 15 |
| GO:0043153 | Entrainment of circadian clock by photoperiod | 1 | 0.32 | 1 |  |  |  |  |  |  | 1 |
| GO:0043010 | Camera-type eye development | 13 | 1 | 1 | 27 | 0.55 | 1 | 41 | 0.0018 | 0.08 | 81 |
| GO:0042752 | Regulation of circadian rhythm | 1 | 0.32 | 1 |  |  |  |  |  |  | 1 |
| GO:0042670 | Retinal cone cell differentiation |  |  |  |  |  |  | 1 | 0.34 | 0.71 | 1 |
| GO:0042573 | Retinoic acid metabolic process |  |  |  | 1 | 0.7 | 1 | 2 | 0.27 | 0.71 | 3 |
| GO:0042461 | Photoreceptor cell development | 2 | 0.84 | 1 | 5 | 0.14 | 0.98 | 2 | 0.87 | 0.96 | 9 |
| GO:0035845 | Photoreceptor cell outer segment organization |  |  |  | 3 | 0.036 | 0.98 |  |  |  | 3 |
| GO:0034653 | Retinoic acid catabolic process |  |  |  | 1 | 0.55 | 1 | 1 | 0.56 | 0.81 | 2 |
| GO:0034644 | Cellular response to UV | 1 | 0.32 | 1 |  |  |  |  |  |  | 1 |
| GO:0031290 | Retinal ganglion cell axon guidance | 2 | 0.97 | 1 | 5 | 0.51 | 1 | 7 | 0.16 | 0.71 | 14 |
| GO:0021634 | Optic nerve formation |  |  |  |  |  |  | 2 | 0.12 | 0.64 | 2 |
| GO:0021631 | Optic nerve morphogenesis |  |  |  |  |  |  | 2 | 0.12 | 0.64 | 2 |
| GO:0021554 | Optic nerve development | 1 | 0.79 | 1 | 1 | 0.8 | 1 | 2 | 0.42 | 0.78 | 4 |
| GO:0019684 | Photosynthesis, light reaction | 3 | 0.4 | 1 |  |  |  | 3 | 0.44 | 0.81 | 6 |
| GO:0016038 | Absorption of visible light | 1 | 0.32 | 1 |  |  |  |  |  |  | 1 |
| GO:0016037 | Light absorption | 1 | 0.32 | 1 |  |  |  |  |  |  | 1 |
| GO:0010842 | Retina layer formation | 1 | 0.95 | 1 | 4 | 0.25 | 0.98 | 3 | 0.55 | 0.81 | 8 |
| GO:0009649 | Entrainment of circadian clock | 1 | 0.32 | 1 |  |  |  |  |  |  | 1 |
| GO:0009648 | Photoperiodism | 3 | 0.099 | 1 |  |  |  | 1 | 0.81 | 0.92 | 4 |
| GO:0009584 | Detection of visible light | 2 | 0.51 | 1 | 2 | 0.53 | 1 | 1 | 0.87 | 0.96 | 5 |
| GO:0009416 | Response to light stimulus | 13 | 0.02 | 1 | 5 | 0.94 | 1 | 6 | 0.88 | 0.96 | 24 |
| GO:0009411 | Response to UV | 4 | 0.039 | 1 |  |  |  | 1 | 0.87 | 0.96 | 5 |
| GO:0008057 | Eye pigment granule organization |  |  |  |  |  |  | 1 | 0.34 | 0.71 | 1 |
| GO:0008020 | G-protein coupled photoreceptor activity | 2 | 0.1 | 0.88 |  |  |  |  |  |  | 2 |
| GO:0007634 | Optokinetic behavior | 2 | 0.38 | 1 | 1 | 0.8 | 1 | 1 | 0.81 | 0.93 | 4 |
| GO:0007632 | Visual behavior | 2 | 0.38 | 1 | 1 | 0.8 | 1 | 1 | 0.81 | 0.93 | 4 |
| GO:0007623 | Circadian rhythm | 2 | 0.51 | 1 | 2 | 0.54 | 1 | 1 | 0.87 | 0.96 | 5 |
| GO:0007602 | Phototransduction | 3 | 0.19 | 1 | 1 | 0.87 | 1 | 1 | 0.87 | 0.96 | 5 |
| GO:0007601 | Visual perception | 8 | 0.19 | 1 | 8 | 0.21 | 0.98 | 2 | 0.99 | 1.00 | 18 |
| GO:0007600 | Sensory perception | 21 | 0.07 | 1 | 16 | 0.57 | 1 | 11 | 0.97 | 1.00 | 48 |
| GO:0007423 | Sensory organ development | 32 | 1 | 1 | 46 | 0.77 | 1 | 70 | 0.00091 | 0.06 | 148 |
| GO:0005212 | Structural constituent of eye lens | 2 | 0.71 | 0.98 | 1 | 0.94 | 1 | 4 | 0.17 | 0.83 | 7 |
| GO:0003913 | DNA photolyase activity | 2 | 0.24 | 0.88 |  |  |  | 1 | 0.7 | 0.95 | 3 |
| GO:0003904 | Deoxyribodipyrimidine photo-lyase activity | 2 | 0.1 | 0.88 |  |  |  |  |  |  | 2 |
| GO:0003407 | Neural retina development | 1 | 0.99 | 1 | 5 | 0.28 | 0.98 | 5 | 0.3 | 0.71 | 11 |
| GO:0003406 | Retinal pigment epithelium development |  |  |  |  |  |  | 4 | 0.013 | 0.26 | 4 |
| GO:0002088 | Lens development in camera-type eye | 2 | 0.84 | 1 | 4 | 0.34 | 0.99 | 3 | 0.64 | 0.87 | 9 |
| GO:0002072 | Optic cup morphogenesis involved in camera-type eye development |  |  |  | 1 | 0.8 | 1 | 3 | 0.12 | 0.64 | 4 |
| GO:0001895 | Retina homeostasis | 2 | 0.51 | 1 | 1 | 0.87 | 1 | 2 | 0.55 | 0.81 | 5 |
| GO:0001754 | Eye photoreceptor cell differentiation | 2 | 0.91 | 1 | 7 | 0.036 | 0.98 | 2 | 0.93 | 0.99 | 11 |
| GO:0001750 | Photoreceptor outer segment | 1 | 0.31 | 1 |  |  |  |  |  |  | 1 |
| GO:0001654 | Eye development | 16 | 1 | 1 | 35 | 0.45 | 1 | 50 | 0.0014 | 0.07 | 101 |
| GO:0001523 | Retinoid metabolic process |  |  |  | 4 | 0.097 | 0.98 | 2 | 0.66 | 0.87 | 6 |
| GO:0000719 | Photoreactive repair | 1 | 0.32 | 1 |  |  |  |  |  |  | 1 |

**Note:** The GO entries marked in red were significantly enriched (FDR < 0.1).

**Table S7.** The expression patterns of visual related genes in the work of *Sinocyclocheilus*, *A. mexicanus*, and cave loach.

| **Gene name** | ***Astyanax mexicanus*** | ***Sinocyclocheilus anshuiensis*** | ***Sinocyclocheilus anophthalmus*** | ***Triplophysa rosa*** |
| --- | --- | --- | --- | --- |
| ***hsp90a*** | up (Rohner et al., 2013) | up (Yang et al., 2016) |  | no different |
| ***pitx3*** | ds mutation (QTL, McGaugh et al., 2014) |  |  | down |
| ***β\|γ-crystallin*** | down (Thanos et al., 2014) | down (Yang et al., 2016) | up (Meng et al., 2013) | down |
| ***crx*** | down (Jeffery et al., 2009) | down (Yang et al., 2016) | down (Meng et al., 2013) | down |
| ***rx1*** | down (Strickler et al., 2002) |  | no different (Meng et al., 2013) | down |
| ***rx2*** |  |  | down (Meng et al., 2013) | no different |
| ***rx3*** | down (McGaugh et al., 2014) |  |  | high dN/dS |
| ***prph2*** | down (Gross et al. 2013) | down (Yang et al., 2016) |  | down |
| ***nrl*** | down (Stahl et al., 2017) | down (Yang et al., 2016) |  |  |
| ***otx2*** | down (Stahl et al., 2017) | down (Yang et al., 2016) |  |  |
| ***otx5*** | down (stricler et al., 2009) | down (Yang et al., 2016) | down (Meng et al., 2013) |  |
| ***nr2e3*** | down (stricler et al., 2009) | down (Yang et al., 2016) | down (Meng et al., 2013) | down |
| ***ggca1a*** | down (stricler et al., 2009) | down (Yang et al., 2016) |  |  |
| ***gnat1*** | down (stricler et al., 2009) | down (Yang et al., 2016) | down (Meng et al., 2013) | down |
| ***gnat2*** | down (stricler et al., 2009) | down (Yang et al., 2016) | down (Meng et al., 2013) | down |
| ***rorb*** |  | down (Yang et al., 2016) |  | down |

**Note.** The genes were mentioned in at least two species.


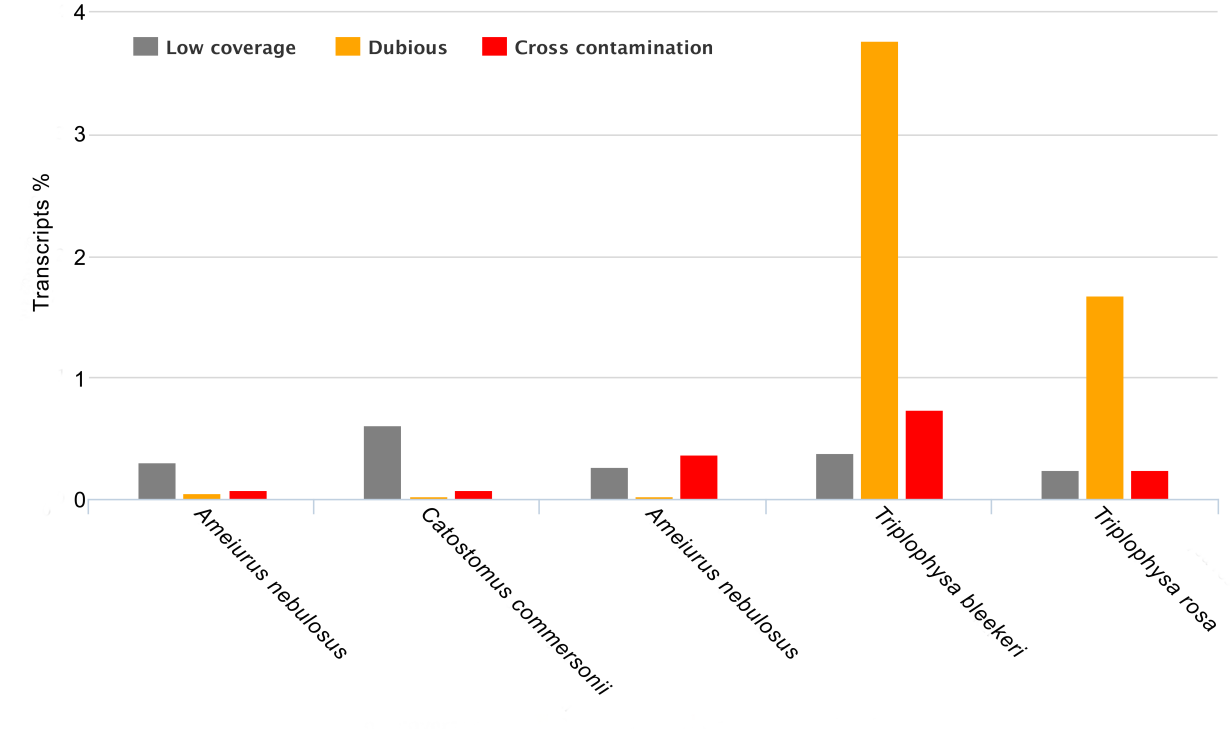


**Figure S1.** The cross contaminations observed in transcriptomic datasets from *Triplophysa* and Hahn et al., 2016. For each transcriptome, three columns indicate the percentage of transcripts categorized as low coverage (grey bars), dubious (orange bars) and cross contamination (red bars) as detected by CroCo (using default parameters). The cross contaminations detected were not applied in *Cobitis taenia* and *Misgurnus anguillicaudatus,* because of the 454 sequencing and single species’ comparative analysis with enough sequence data.


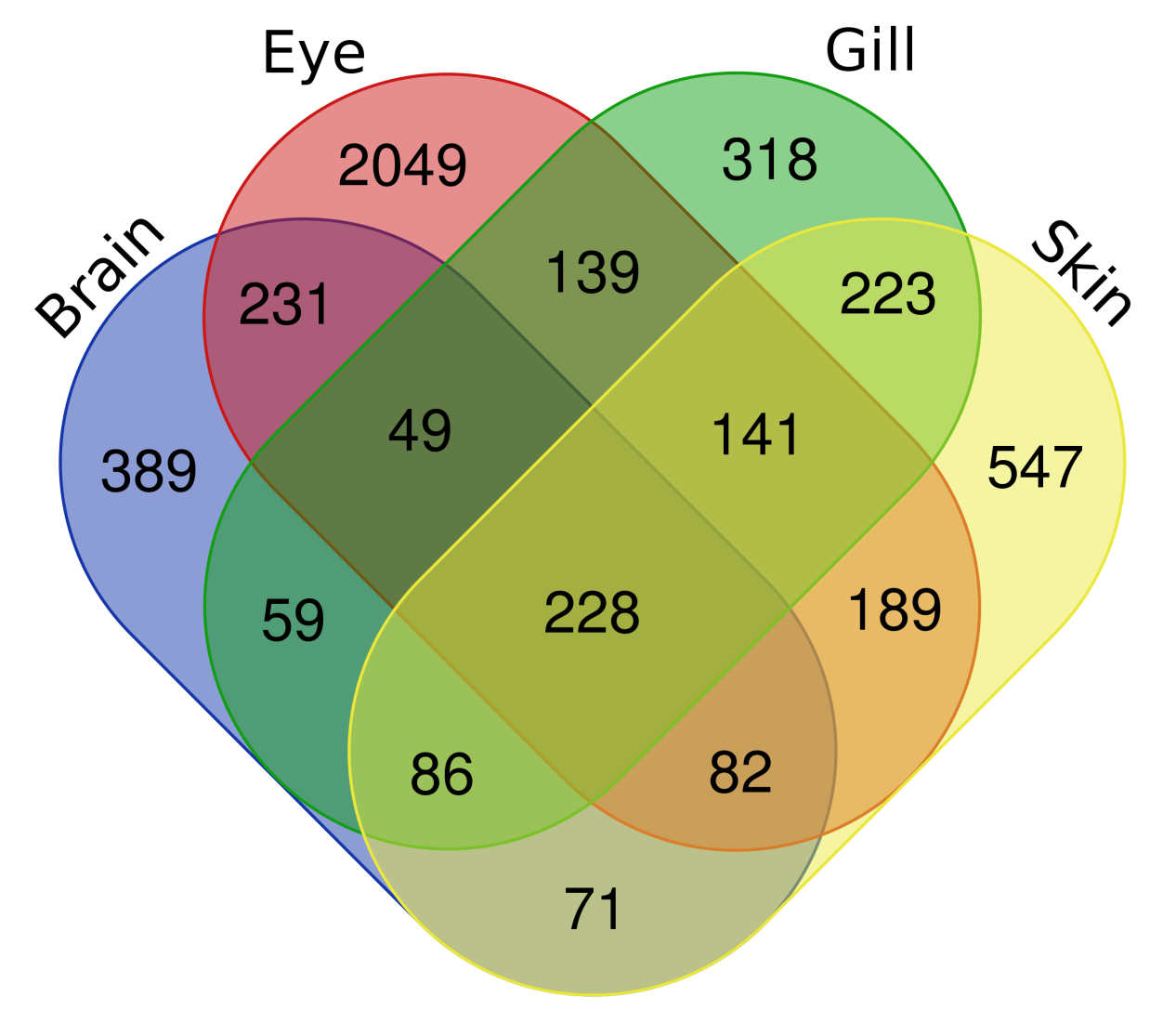


**Figure S2.** Venn diagram of the DEGs involve in four tissues.


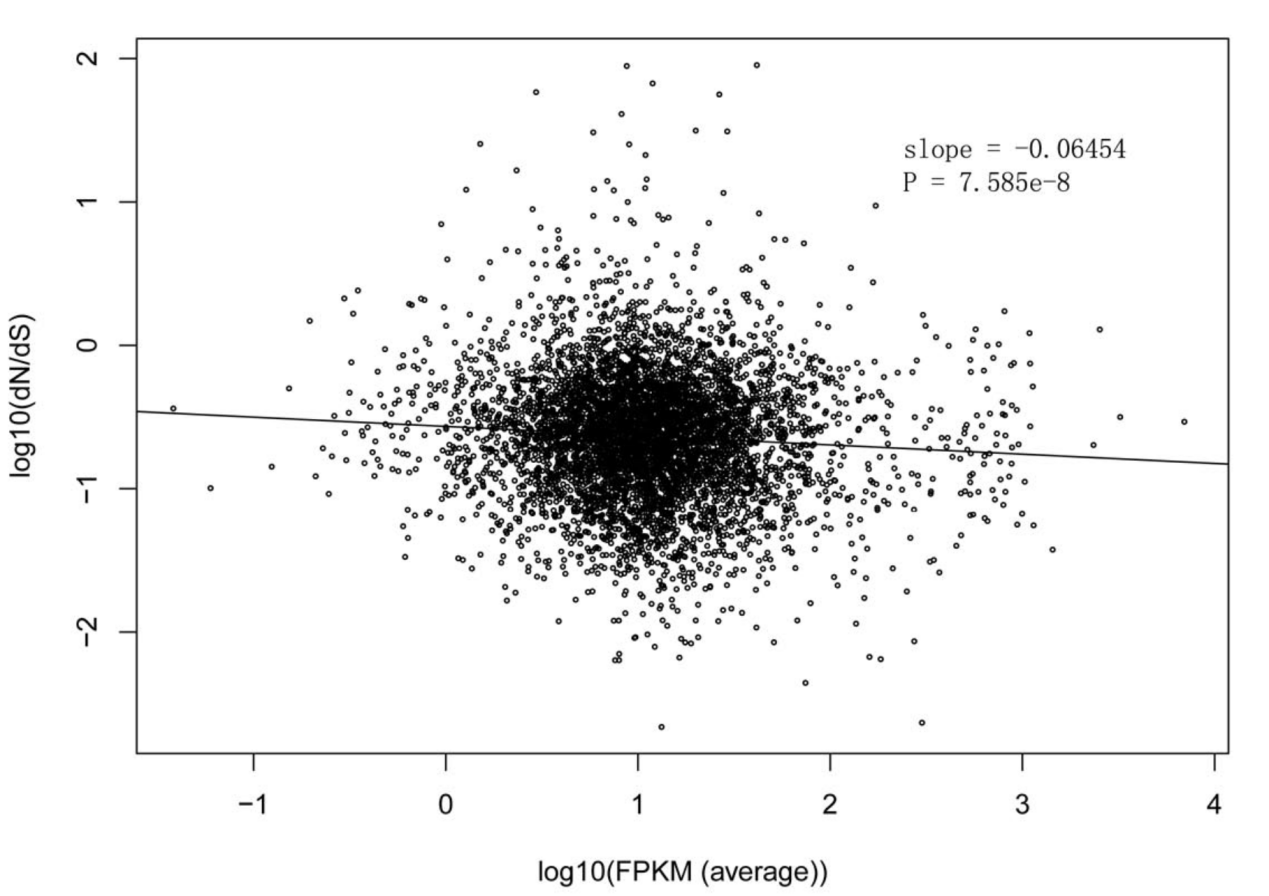


**Figure S3.** Average gene expression level is negative correlated with the dN/dS rate; p-value determined by linear regression.


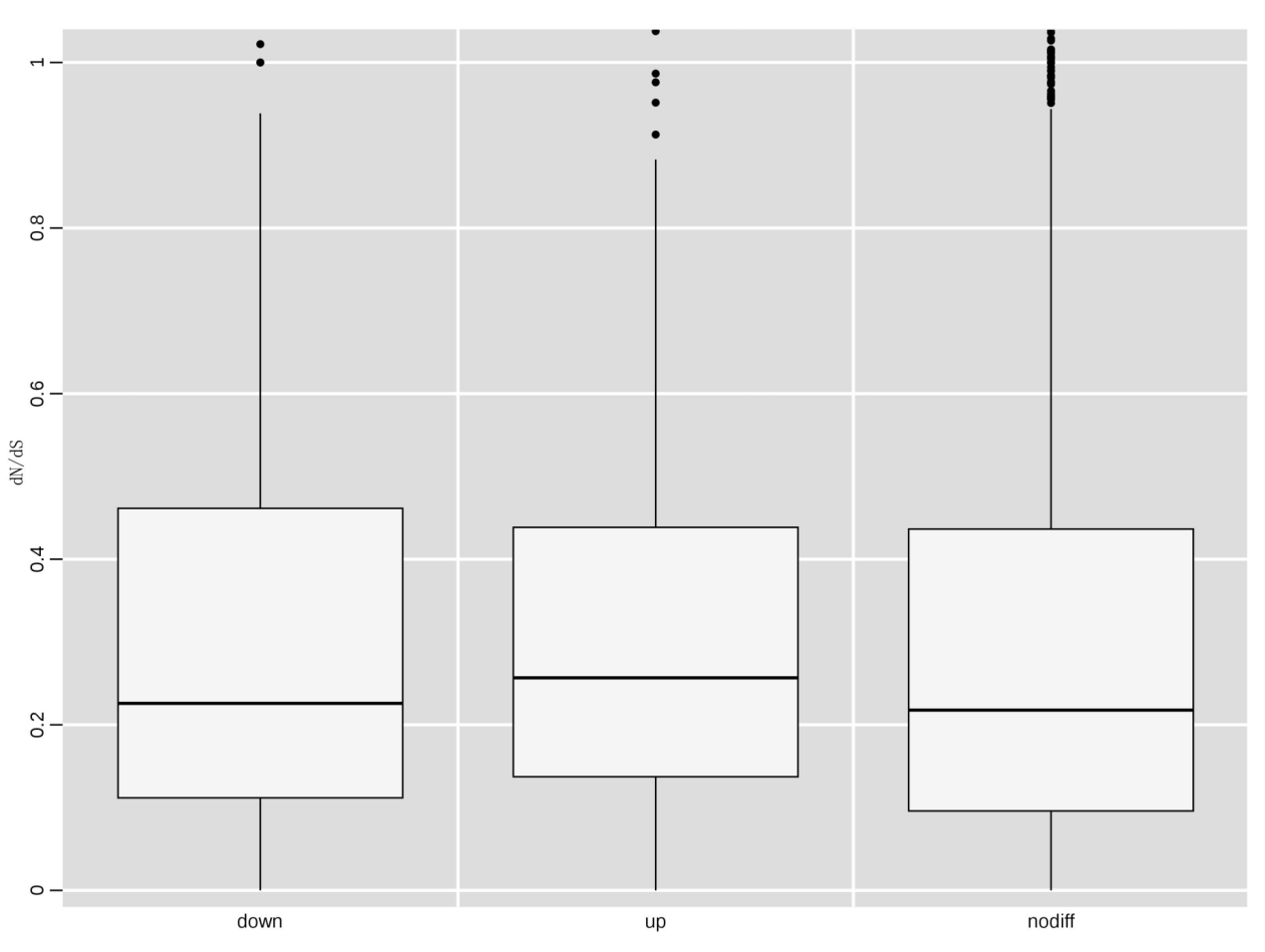


**Figure S4.** The dN/dS value of genes up regulated is no different with genes down regulated in *T. rosa* eyes (tested by Two-tailed T test). down: down regulated genes; up: up regulated genes; nodiff: the genes show no different in expression between *T. rosa* and *T. bleekeri*.


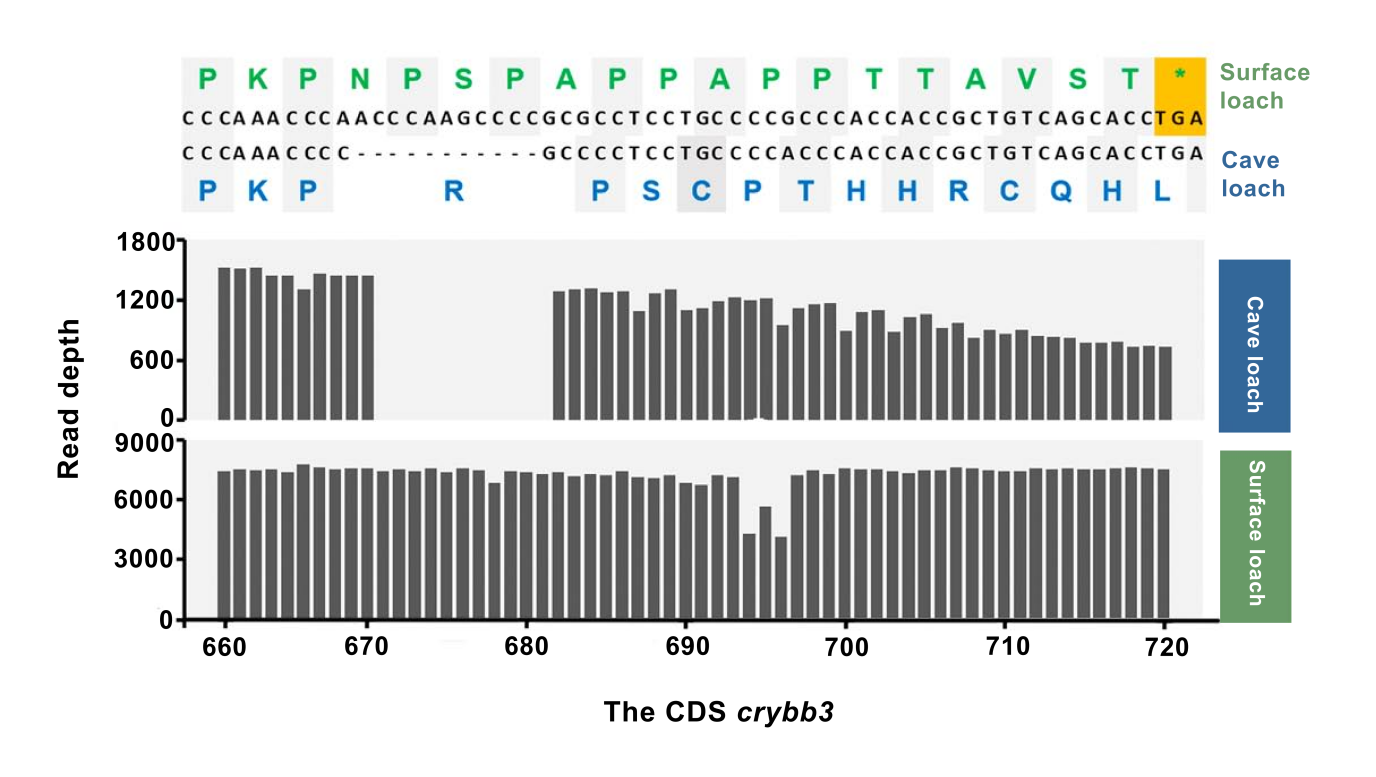


**Figure S5.** Alignment of nucleotide and amino acid sequences (top) and sequencing read depth (bottom; the numbers along the x-axis represent the position of the base at the CDS) for the *crybb3* gene. The translation may not be terminated normally in cave loach due to the generation of a frameshift mutation in *crybb3* mRNA.
